# Ectopic expression of *Medicago truncatula* homeodomain finger protein, *MtPHD6*, enhances drought tolerance in Arabidopsis

**DOI:** 10.1186/s12864-019-6350-5

**Published:** 2019-12-16

**Authors:** Wenli Quan, Xun Liu, Lihua Wang, Mingzhu Yin, Li Yang, Zhulong Chan

**Affiliations:** 1 Key Laboratory for Quality Control of Characteristic Fruits and Vegetables of Hubei Province, College of Life Science and Technology, Hubei Engineering University, Xiaogan, Hubei China; 2 Institute of Molecular Physiology and Biotechnology of Plants (IMBIO), University of Bonn, Bonn, Germany; 3 College of Basic Medicine, Hubei University of Chinese Medicine, Wuhan, Hubei China; 4 Key Laboratory of Horticultural Plant Biology, Ministry of Education; Key Laboratory of Urban Agriculture in Central China, Ministry of Agriculture; College of Horticulture and Forestry Sciences, Huazhong Agricultural University, Wuhan, Hubei China; 5 Wuhan Botanical Garden, Chinese Academy of Sciences, Wuhan, Hubei China

**Keywords:** ABA, Drought stress, Interaction network, *Medicago truncatula*, PHD finger protein, Zinc finger protein

## Abstract

**Background:**

The plant homeodomain (PHD) finger is a Cys_4_HisCys_3_-type zinc finger which promotes protein-protein interactions and binds to the *cis*-acting elements in the promoter regions of target genes. In *Medicago truncatula*, five *PHD* homologues with full-length sequence were identified. However, the detailed function of *PHD* genes was not fully addressed.

**Results:**

In this study, we characterized the function of *MtPHD6* during plant responses to drought stress. *MtPHD6* was highly induced by drought stress. Ectopic expression of *MtPHD6* in Arabidopsis enhanced tolerance to osmotic and drought stresses. *MtPHD6* transgenic plants exhibited decreased water loss rate, MDA and ROS contents, and increased leaf water content and antioxidant enzyme activities under drought condition. Global transcriptomic analysis revealed that *MtPHD6* reprogramed transcriptional networks in transgenic plants. Expression levels of ABA receptor *PYR/PYL*s, *ZINC FINGER*, *AP2/EREBP* and *WRKY* transcription factors were mainly up-regulated after transformation of *MtPHD6*. Interaction network analysis showed that ZINC FINGER, AP2/EREBP and WRKY interacted with each other and downstream stress induced proteins.

**Conclusions:**

We proposed that *ZINC FINGER*, *AP2*/*EREBP* and *WRKY* transcription factors were activated through ABA dependent and independent pathways to increase drought tolerance of *MtPHD6* transgenic plants.

## Background

The plant homeodomain (PHD) finger was named according to the Arabidopsis Histone acetyltransferases 3.1 (HAT3.1) [1]. As a common structural motif, it is found in all eukaryotic genomes and typically shows a Cys_4_HisCys_3_-type zinc finger signature [2, 3]. Along with promoting protein-protein interactions, the PHD-finger motif can also bind to the *cis*-acting elements in the promoter regions of target genes [4]. It has been widely reported that PHD-finger-containing proteins are localized in nucleus and most likely to be chromatin-mediated transcriptional regulators [3, 5]. In plants, many studies have shown that PHD-finger-containing proteins are involved in diverse physiological processes, including the epigenetic silencing, regulation of the flowering time, and the growth and development of roots [6–9].

Alfin1 from *Medicago sativa* (alfalfa) is a member of plant-specific PHD-finger protein subfamily. It was shown that alfin1 is a salt-inducible transcription factor and can regulate the expression of *MsPRP2* gene, leading to enhanced salt tolerance [4, 10]. Alfin1-like (AL) proteins belonging to a small group of PHD-finger proteins were originally discovered to be a kind of transcription factor family in alfalfa [4]. Recently, more and more *AL* genes have been reported in many different plant species, including *Arabidopsis thaliana*, *Oryza sativa*, *Glycine max*, *Brassica rapa*, *Brassica oleracea*, *Zea mays* and *Atriplex hortensis* [5, 7, 11–15]. The expression of these genes is stress-responsive and varies due to stress types [7, 12]. Six *GmPHD* genes were identified from soybean and encoded Alfin1-type PHD finger proteins, which could response to diverse abiotic stresses. Ectopic expression of *GmPHD2* in Arabidopsis improved salt tolerance of transgenic plants with decreased reactive oxygen species (ROS) [5]. GmPHD5 was capable of regulating histone crosstalk between methylated H3K4 and acetylated H3K14 in response to salinity stress, and could also recruit chromatin remodeling factors and salt stress induced transcription factors such as *GmRD22* and *GmGST* to regulate their expression levels [16]. PHD fingers in ING (inhibitor of growth) homologues and AL proteins in Arabidopsis could bind to H3K4me3/2, the active histone markers in plants [6, 17]. AtAL6 played crucial roles in phosphate deficiency-induced root hair formation by binding to H3K4me3 of ETC1 through the PHD domain [6]. T-DNA insertion mutant and overexpression of *AtAL7* exhibited a negative role in salt tolerance of *A. thaliana* [15]. *AtAL5–*overexpressing plants showed improved tolerance to salt, drought and cold stress [18]. Ectopic expression of *AhAL1* contributed to improving survival rates of transgenic Arabidopsis plants under salt and drought conditions [13]. Above evidences suggested that *AL/PHD* genes play important roles in regulating plant responses to abiotic stresses by changing transcription and reading epigenetic histone modifications.

Forages are key components of sustainable agriculture. As a model plant of forage legume species, *M. truncatula* has been widely studied at the molecular level [19]; and has a close relationship to alfalfa which is the world’s most important forage legume [20]. However, forages are often grown in relatively severe environmental conditions, resulting in evolution of complicated protective mechanisms for survival [21]. Drought is the main environmental factor that limits plant growth, development and productivity in arid and semiarid regions [22]. Hence, the tolerance of drought is a vital breeding trait for forage legume. To date, the function of several transcription factors from *M. truncatula* has been dissected in plant responses to drought stress, including *WXP1* and *SPL8* [19, 23]. Overexpression of *MtWXP1* in alfalfa facilitated plants to accumulate cuticular wax and improved the tolerance of drought [19]. However, the detailed function of *MtPHDs* in response to drought stress remains elusive.

In the present study, expression of *MtPHD6* by drought stress was investigated. Drought tolerance of *MtPHD6* transgenic Arabidopsis was identified in the physiological level. Water loss, oxidative damage and antioxidant enzyme activities were assayed. Genome-wide transcriptomic analysis of *MtPHD6* transgenic plants under drought stress was performed to identify *MtPHD6* mediated genes. Additionally, protein interaction networks modulated by *MtPHD6* transgene were characterized. Our results provide further insights into the roles and the molecular mechanisms of *MtPHD6* in regulating plant responses to drought stress.

## Results

### Characterization of *MtPHDs* family in *M. truncatula*

**Fig. 1:**
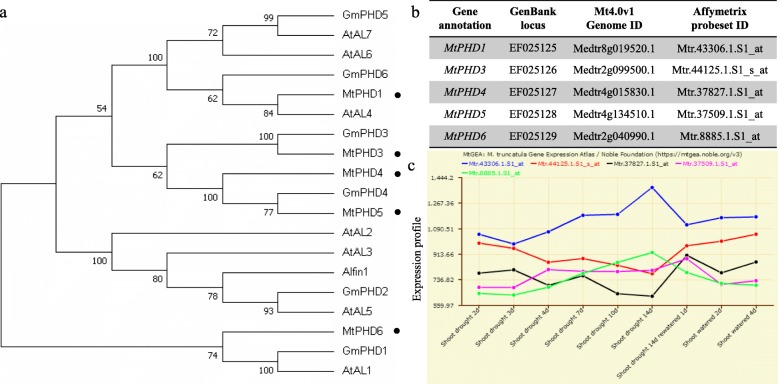
Phylogenic tree of PHD-finger proteins from different species and expression analysis of *MtPHDs* under drought stress*.* **a**: The phylogenic tree was constructed using the neighbor-joining method in MEGA 7.0 software with 1000 bootstrap. Numbers on the figure are bootstrap values. The protein sequences are from *Medicago truncatula* (MtPHDs); *Medicago sativa* (Alfin1); *Glycine max* (GmPHDs); *Arabidopsis thaliana* (AtALs). Accession numbers were listed in Additional file 1: Table S1. **b**: Information of *MtPHDs*. **c**: Expression changes of *MtPHDs* under drought stress condition. The data are available from the *M. truncatula* Gene Expression Atlas (MtGEA) web server at https://mtgea.noble.org/v3/

In *M. truncatula*, seven *PHD* homologues were identified and five full-length sequences were termed *MtPHD*1 and 3–6 (EF025125, EF025126, EF025127, EF025128, and EF025129) [5], among which MtPHD5 was almost identical to Alfin1 of *M. sativa* [4, 24]. Phylogenetic analysis of these five MtPHDs with ALs/PHDs from other plant species showed that MtPHDs were clustered into five different clades. However, MtPHD6 (EF025129/Medtr2g040990) was most distantly related to the other members of the MtPHDs family, and was more closely related to GmPHD1 and AtAL1 (Fig. 1a), which were significantly induced by PEG/drought and NaCl treatments [5, 18]. The detailed information of *MtPHDs* with Mt4.0 V1 genome (http://www.medicagogenome.org/home) and Affymetrix probeset IDs were presented (Fig. 1b). Affymetrix microarray data showed that *MtPHD6* (Mtr.8885.1.S1_at) increased upon drought stress treatment, but decreased after re-watering (Fig. 1c).

### *MtPHD6*-overexpressing plants showed increased tolerance to osmotic stress

**Fig. 2:**
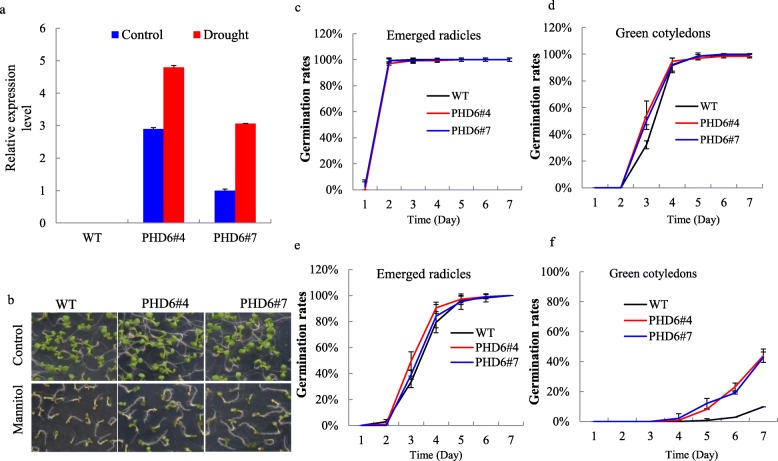
Expression changes of *MtPHD6* under drought stress and osmotic stress tolerance of *MtPHD6* transgenic Arabidopsis. **a**: The relative expression level of *MtPHD6* after drought 7 d for 12-day-old transgenic seedlings through qRT-PCR; **b**: Phenotypic changes after 300 mM mannitol treatment; **c**, **e**, Emerged radicles under control and osmotic stress conditions, respectively; **d**, **f**: Green cotyledons under control and osmotic stress conditions, respectively

To test the function of *MtPHD6* in response to abiotic stresses, *MtPHD6* gene was cloned from *M. truncatula* using specific primers listed in Additional file 2: Table S2 and transgenic Arabidopsis plants over-expressing *MtPHD6* were constructed. Drought treatment indeed significantly up-regulated the *MtPHD6* expression in two transgenic lines (*PHD6*#4 and *PHD6*#7) when compared to control condition (Fig. 2a). These results showed that *MtPHD6* is stress-inducible and might play roles in plant responses to drought stress.

Under 300 mM mannitol condition, *MtPHD6* transgenic plants exhibited more vigorous growth than WT (Fig. 2b). There were no significant differences of emerged radicles and green cotyledons between transgenic lines and WT under normal growth condition (Fig. 2c, d). After 300 mM mannitol treatment for 7 d, the transgenic lines showed significantly higher percentages of green cotyledons (Fig. 2f), although emerged radicles between transgenic lines and WT had no significant differences (Fig. 2e). The results indicated that *MtPHD6* might play a positive role in response to osmotic stress during seed germination stage.

**Fig. 3:**
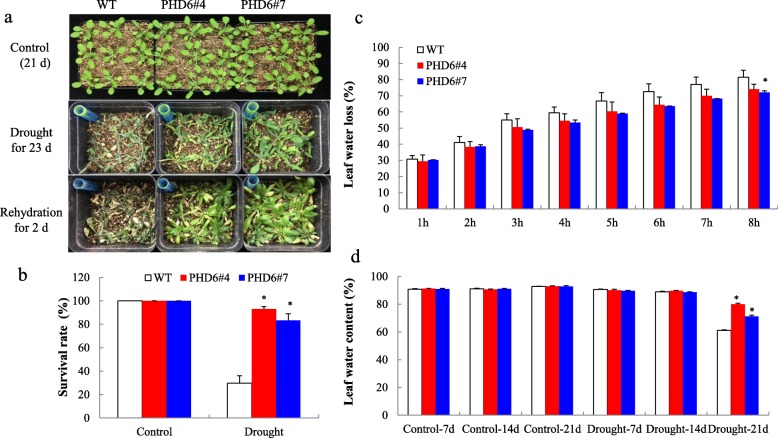
Drought stress tolerance of *MtPHD6* transgenic lines. **a**: Phenotypic changes after drought treatment. 12-day-old seedlings were withheld water for 23 days. Photos were taken at 2 d after rehydration; **b**: Survival rate of *MtPHD6* transgenic lines and WT after 2 d rehydration; **c**: Leaf water loss of *MtPHD6* transgenic lines and WT. 21-day-old leaves were detached and air-dried for up to 8 h. **d**: Leaf water content of *MtPHD6* transgenic lines and WT after 7, 14, 21 d drought treatment

In order to further investigate drought tolerance, 12-day-old plants from normal growth condition were water-withheld for 23 d, and then the plants were re-watered for 2 d. The results showed that WT plants suffered more serious withering and damage from drought treatment (Fig. 3a). After rehydration, fewer than 30% of WT plants were alive whereas more than 80% of transgenic plants survived (Fig. 3b). Under control condition, the leaf water loss of transgenic lines was lower than that of WT (Fig. 3c). When subjected to drought treatment, transgenic plants showed significantly higher leaf water content than WT plants at drought 21 d (Fig. 3d). Taken together, these results suggested that *MtPHD6* transgenic plants showed improved tolerance to drought stress through the regulation of water loss.

### Transcriptomic profiling analysis of *MtPHD6* ectopic expression plants after drought treatment

To characterize molecular mechanisms of *MtPHD6*-modulated drought tolerance, we performed RNA-sequencing to identify DEGs affected by the *MtPHD6* transgene and drought treatment. In this study, twelve samples with three biological replicates per genotype/treatment combination were used. Each sample with at least 6 G clean data was obtained. In total, 2044 genes were transcriptionally affected by the *MtPHD6* transgene or drought treatment (Additional file 3: Table S3). Quantitative real-time PCR qRT-PCR analysis showed that the trends of both up-regulated and down-regulated expression measured by RNA-seq and by qRT-PCR were similar (Additional file 7: Figure S1), indicating RNA-sequencing data were reliable.

**Fig. 4:**
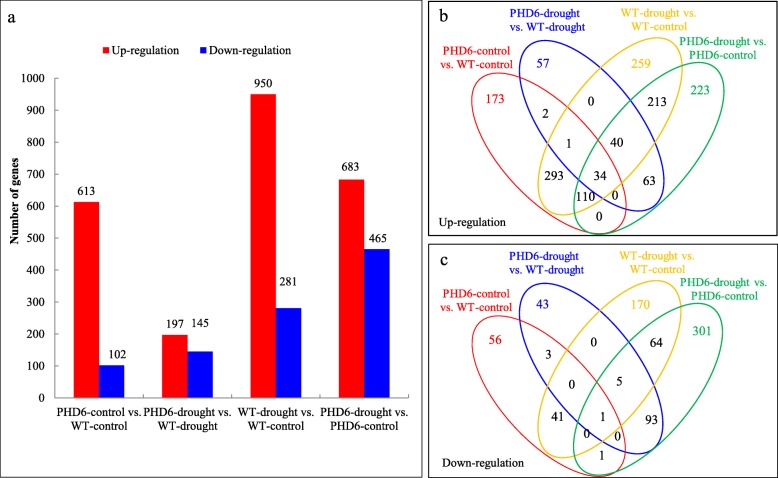
*MtPHD6* transgene- and drought stress-modulated genes through RNA-seq analysis. **a**: Number of genes changed by *MtPHD6* transgene or drought stress. **b** and **c**: Overlapping analysis of up- and down-regulated genes by *MtPHD6* transgene or drought stress. Arabidopsis seedlings at 12-day-old were subjected to drought treatment for 7 days. Leaves of transgenic lines and WT plants were harvested for RNA-seq analysis. The original data were presented in Additional file 3: Table S3

Bioinformatics analysis showed that *MtPHD6* transgene affected expression level of 715 and 342 genes under control and drought stress conditions, respectively. In WT plants, drought stress treatment modulated expression of 1231 genes, including 950 up-regulated and 281 down-regulated genes. Comparatively, significantly fewer DEGs were identified by drought stress treatment in *MtPHD6* OE lines, with 683 up-regulated genes and 465 down-regulated genes (Fig. 4a). Overlapping analysis indicated that there were 397 up-regulated and 70 down-regulated genes which were commonly regulated in both *MtPHD6* OE lines and WT plants after drought treatment (*PHD6*-drought vs. *PHD6*-control; WT-drought vs. WT-control), respectively. Moreover, 438 up-regulated and 42 down-regulated genes were commonly regulated by *MtPHD6* transgene under control condition and drought stress condition (*PHD6*-control vs. WT-control; WT-drought vs. WT-control) (Fig. 4b, c).

### Cluster, GO term enrichment and MapMAN pathway analyses

**Fig. 5:**
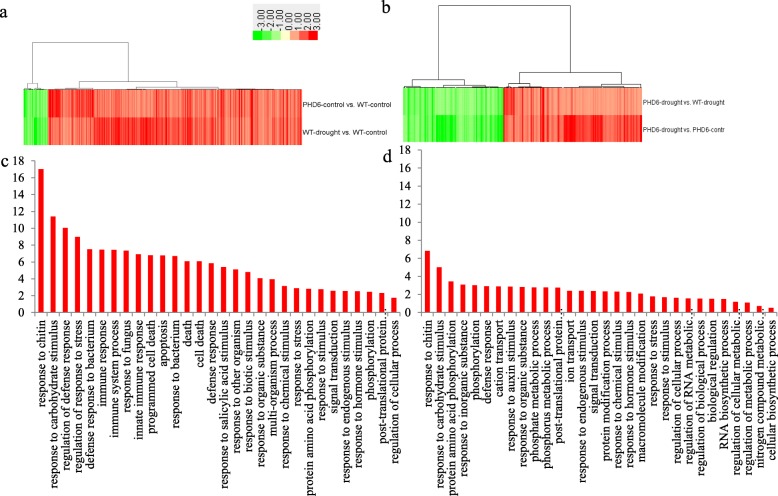
Cluster and GO term enrichment analyses of genes commonly regulated by *MtPHD6* transgene and drought stress. In total 480 genes were commonly modulated by *PHD6*-control vs. WT-control and WT-drought vs. WT-control (**a**), while 236 genes were commonly modulated by *PHD6*-drought vs. WT-drought and *PHD6*-drought vs. *PHD6*-control (**b**). The lists of genes were submitted to agriGO (http://bioinfo.cau.edu.cn/agriGO/) with the analysis tool “Singular Enrichment Analysis (SEA)”. Top 15 significantly enriched GO terms with lowest *P*-value were listed (**c** and **d**). The original data were presented in Additional file 4: Table S4

Totally, 480 genes were commonly regulated by *MtPHD6* transgene under control condition (*PHD6*-control vs. WT-control) and drought stress on WT (WT-drought vs. WT-control), while 236 genes were commonly modulated by *MtPHD6* transgene under drought condition (*PHD6*-drought vs. WT-drought) and drought stress on *MtPHD6* OE lines (*PHD6*-drought vs. *PHD6*-control) (Fig. 5a, b). Further analysis showed that stress, carbohydrate metabolism, protein modification, signal transduction and hormone related GO terms were significantly enriched (Fig. 5c, d).

**Fig. 6:**
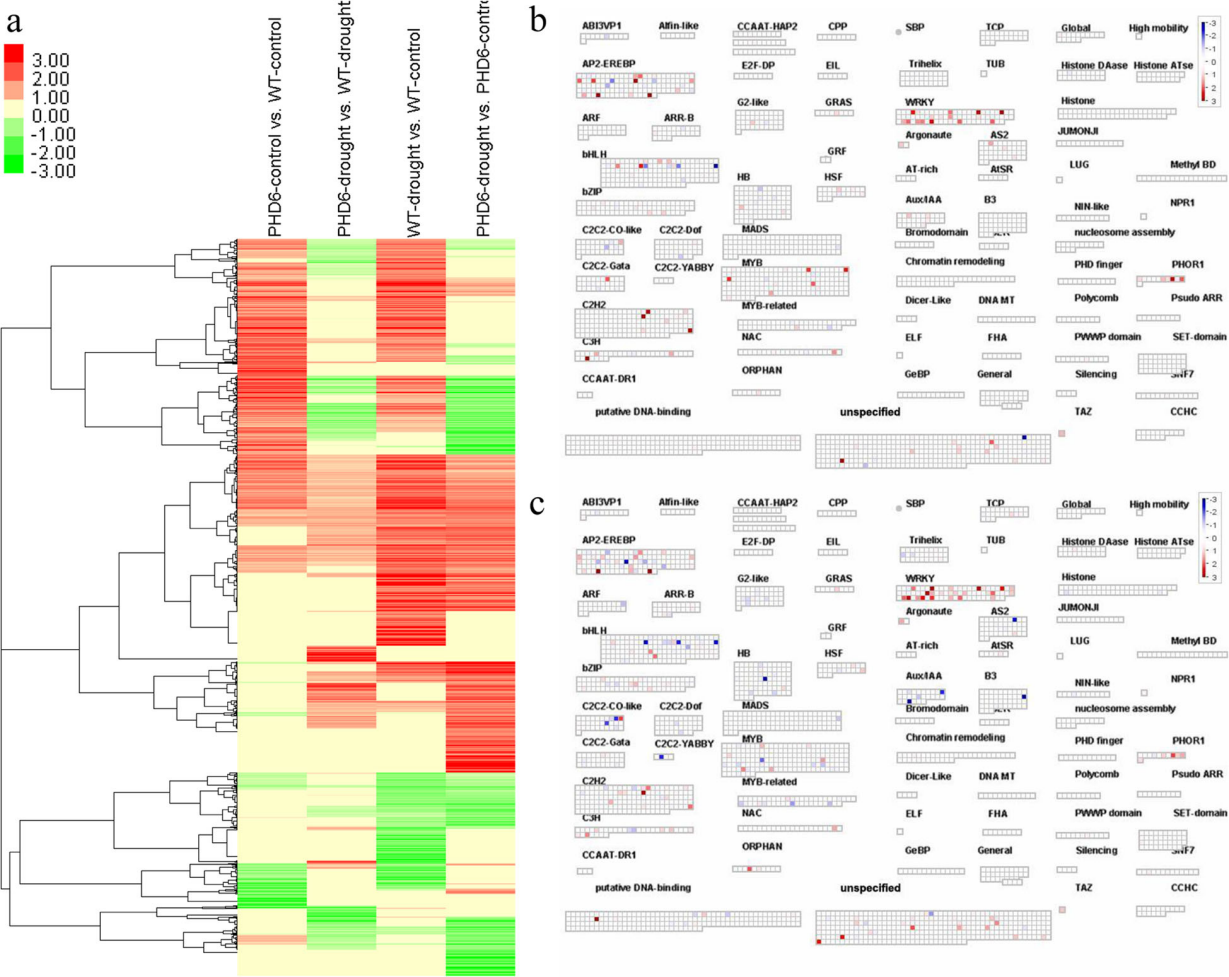
Gene cluster analysis and transcription factors changed by*MtPHD6* transgene or drought treatment. **a**: Cluster analysis of all genes modulated by *MtPHD6* transgene or drought stress; **b** and **c**: Transcription factors regulated by *MtPHD6* transgene or drought stress. Red squares represent up-regulated genes; blue squares mean down-regulated genes; and white squares mean no significant changes. The original data were presented in Additional file 6: Table S6

For all DEGs modulated by *MtPHD6* transgene or drought stress, cluster analysis further revealed that the majority of *MtPHD6* transgene modulated genes were also changed by drought stress (Fig. 6a, columns 1 and 3). Pathway analysis exhibited that many *AP2*/*EREBP*, *WRKY*, and *ZINC FINGER* transcription factors were regulated by *MtPHD6* transgene or drought stress (Fig. 6b, c). DEGs modulated by the *MtPHD6* transgene or drought stress treatment were selected for GO term enrichment analysis. For biological process GO terms, response to stress, signal transduction, response to abiotic or biotic stimulus and other biological processes were significantly enriched (Additional file 8: Figure S2a). For molecular function GO terms, receptor binding or activity, transcription factor activity and kinase activity were significantly enriched (Additional file 8: Figure S2b). For cellular component GO terms, cell wall, plasma membrane and extracellular were significantly enriched (Additional file 8: Figure S2c).

In addition, pathway enrichment analysis was performed for genes affected by *MtPHD6* transgene or drought stress using MapMAN software. The results showed that five pathways were overrepresented by all comparisons, including hormone metabolism, minor CHO metabolism, signaling, cell wall, and stress. Pathway of redox was enriched after drought treatment in both WT and *MtPHD6* transgenic lines, and by *MtPHD6* transgene under control condition (*P* = 0.072) (Table 1). These data indicated that drought stress re-programed transcriptional networks in Arabidopsis and ectopic expression of *MtPHD6* also extensively altered transcriptional networks in Arabidopsis.

**Table 1:**
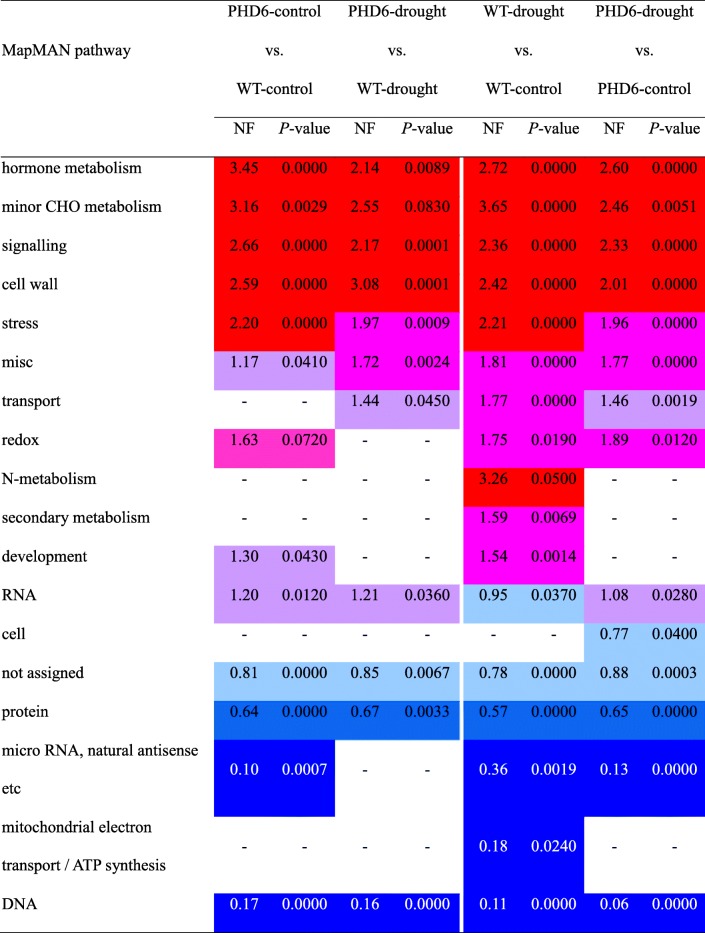
MapMAN pathway enrichment analysis of genes affected by *MtPHD6* transgene or drought stress. DEGs (fold change ≥2 and *P*-value ≤0.05) were annotated using the Classification SuperViewer Tool. MapMAN was used as the classification source. The original data were presented in Additional file 5: Table S5.The scales of normalized frequency (NF) are as follows

### Transcription factors modulated by *MtPHD6* transgene and drought treatment

**Fig. 7:**
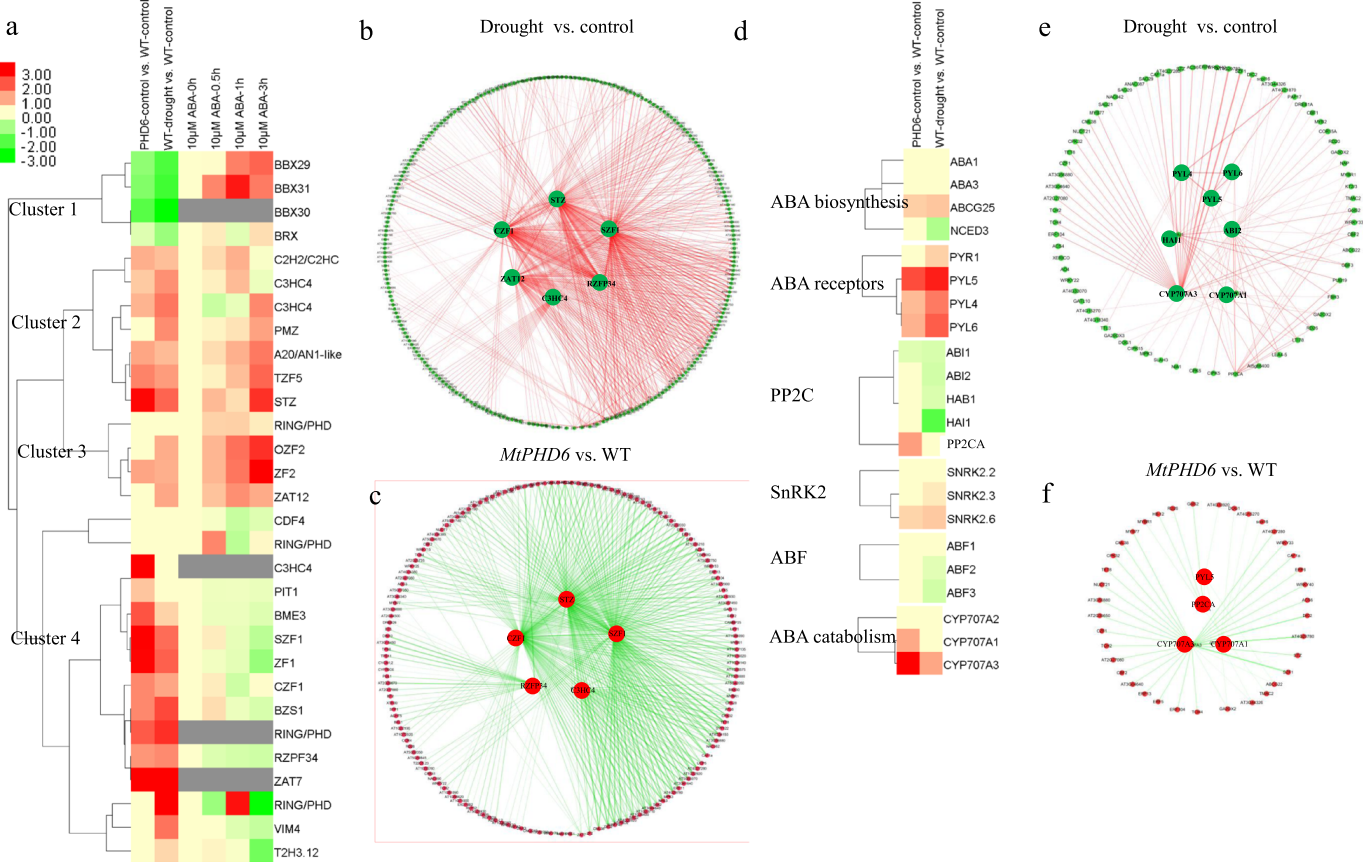
Cluster and network analyses of zinc finger transcription factors and ABA pathway related genes. **a**: Cluster analysis of zinc finger transcription factors affected by *MtPHD6* transgene, drought and ABA. **b** and **c**: Networks of zinc finger transcription factors modulated by drought stress (green node and red edge) and *MtPHD6* transgene (red node and green edge), respectively; **d**: Cluster analysis of ABA signaling pathway related genes affected by *MtPHD6* transgene and drought. **e** and **f**: Networks of ABA signaling pathway related genes modulated by drought stress (green node and red edge) and *MtPHD6* transgene (red node and green edge), respectively. The original data were presented in Additional file 6: Table S6. ABA treatment expression data were downloaded from BAR Expression Browser (http://bar.utoronto.ca/affydb/cgi-bin/affy_db_exprss_browser_in.cgi). The grey color in cluster analysis indicates the genes are missing in BAR database

To identify transcription factors affected by *MtPHD6* transgene and drought stress, we further performed MapMAN pathway analysis. The results showed that *MtPHD6* transgene or drought stress modulated expression of many *AP2*/*EREBP* (*AP2*, *RAV* and *CBF*/*DREB2*/*ERF*), *WRKY*, and *ZINC FINGER* transcription factors (Fig. 6b, c), which have been reported to be involved in plant stress responses [25–27]. In this study, both drought stress and *MtPHD6* transgene modulated expression of several *ZINC FINGER* transcription factors (*STZ*, *SZF1*, *CZF1*, etc) (Fig. 7). Cluster analysis showed that *TZF5*, *STZ*, *OZF2*, *ZF2*, *ZAT12* identified in this study were also upregulated after ABA treatment (Fig. 7a, Clusters 2 and 3). Meanwhile, *BBX29* and *BBX31* were inhibited by drought stress and *MtPHD6* transgene, but were highly induced after ABA treatment (Fig. 7a, Cluster 1). Interaction network analysis revealed that there were 249 nodes/proteins and 513 edges for drought, and 163 nodes/proteins and 388 edges for *MtPHD6* transgene modulated *ZINC FINGER* transcription factors (Fig. 7b, c), including CPK, MPK, ERF, WRKY, DREB/CBF and NAC proteins.

Drought treatment and *MtPHD6* transgene activated expression of the majority of *AP2/EREBP* transcription factors (Additional file 9: Figure S3a). *AP2*/*EREBP* transcription factors in clusters 1 and 2 were mainly downregulated by *MtPHD6* transgene, but upregulated by ABA treatment. However, *AP2*/*EREBP* transcription factors in clusters 4 and 5 were upregulated by *MtPHD6* transgene, but mainly downregulated by ABA treatment (Additional file 9: Figure S3a). These data indicated that *AP2*/*EREBP* transcription factors showed contrasting changes by *MtPHD6* transgene and ABA. Interaction network analysis showed that there were 194 nodes/proteins and 445 edges for drought, and 155 nodes/proteins and 424 edges for *MtPHD6* transgene modulated AP2/EREBP transcription factors (Additional file 9: Figure S3b, c), including WRKY, CML, MYB and ZINC FINGER proteins. WRKY transcription factors were mainly induced by *MtPHD6* transgene and drought, but merely by ABA treatment (Additional file 9: Figure S3d). Interaction network analysis showed that there were 279 nodes/proteins and 807 edges for drought, and 196 nodes/proteins and 630 edges for *MtPHD6* transgene modulated transcription factors (Additional file 9: Figure S3e, f), including MPK, MYB, AP2/ERF and ZINC FINGER proteins.

### ABA pathway regulated by *MtPHD6* transgene and drought treatment

Since zinc finger and AP2/EREBP transcription factors were modulated by ABA treatment based on publicly available microarray data analysis (Fig. 7a), we further investigated the gene expressions of ABA signaling transduction pathway. The results exhibited that several ABA receptor genes were highly induced by drought and *MtPHD6* transgene, especially *PYL4–6*. Meanwhile, *MtPHD6* transgene also activated expression of *PP2CA* and *CYP707A1* and *A3*, while drought stress inhibited expressions of *ABA1*, *ABA2*, *HAB1* and *HAI1*, and induced *CYP707A3* expression (Fig. 7d). To better understand the effect of *MtPHD6* transgene on ABA pathway, the possible interaction networks were also constructed and co-expressions of ABA pathway responsive genes were identified using STRING and Cytoscape software. The results showed that drought stress modulated *PYLs* (*PYL4*–5) and *PP2C* (*HAI1, ABI2, PP2CA*) interacted with several stress responsive genes, including *ERFs*, *DREB1*/*CBF*, *LTI*, *COR*, *RD*, *LEA*, *WRKY*, *NAC* and *ZINC FINGER* transcription factors (*STZ*, *RHL41*/*ZAT12*) (Fig. 7e). In *MtPHD6* transgenic lines, PYLs and PP2Cs interact with ERF, DREB/CBF, RD, and ZINC FINGER transcription factors (STZ, SZF1, CZF1) (Fig. 7f). These results indicated that *MtPHD6* transgene might activate *ZINC FINGER* and *AP2/EREBP* transcription factors through ABA dependent pathway, and induced *WRKY* transcription factors through ABA independent pathway.

### Metabolism pathways regulated by *MtPHD6* transgene and drought stress

**Fig. 8:**
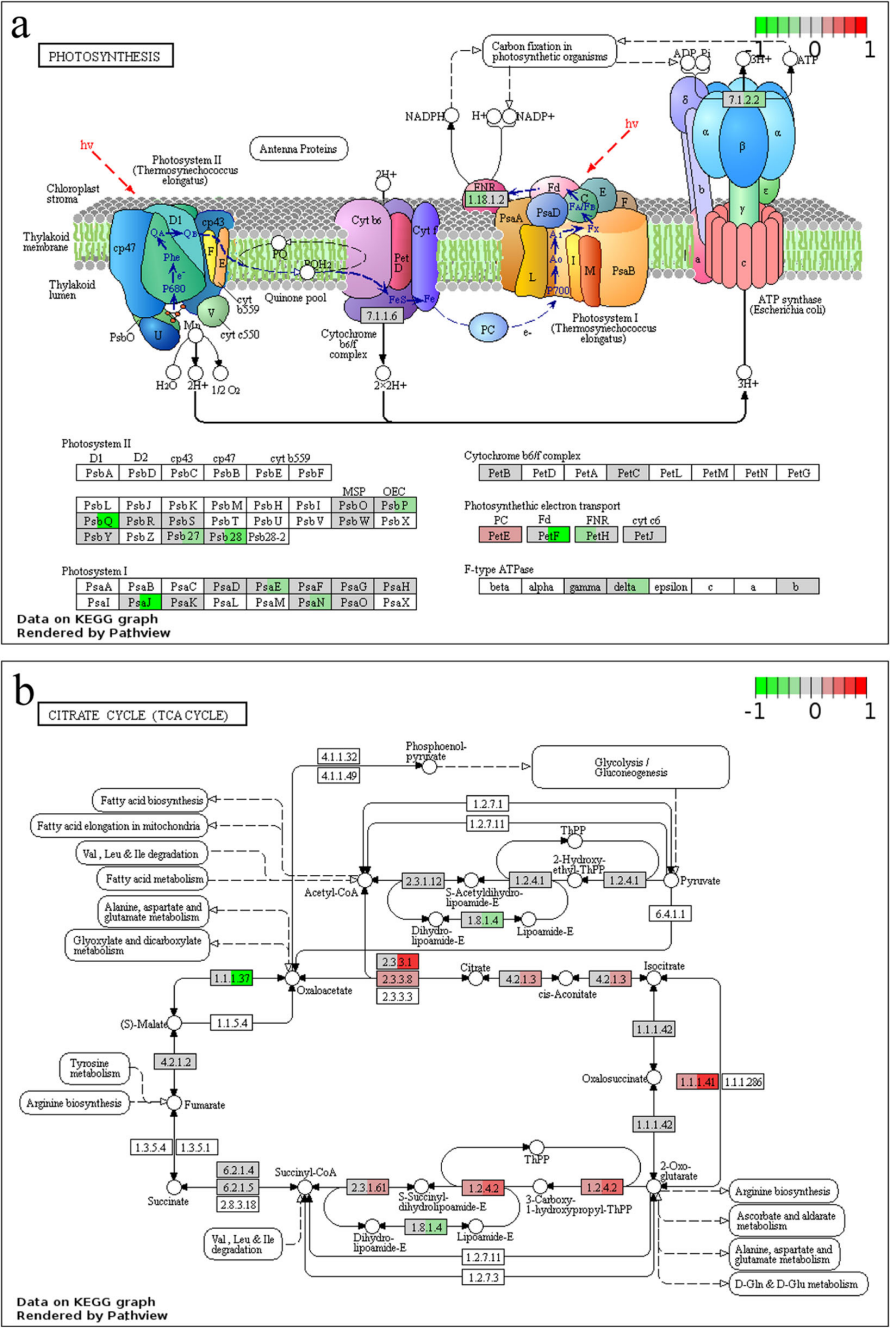
Photosynthesis (**a**) and TCA cycle (**b**) related genes changed by *MtPHD6* transgene or drought. KEGG pathway changes were analyzed using Pathview (https://pathview.uncc.edu/home). Each rectangle was divided into 2 subsections, which represent genes modulated by *MtPHD6* transgene (*MtPHD6*-control vs. WT-control) and drought (WT-drought vs. WT-control), respectively. Red means upregulation and green means downregulation

In the study, drought stress inhibited expression of several genes involved in photosystem I, II and photosynthetic electron transport pathways (Fig. 8a). However, few of photosynthesis related genes were regulated by *MtPHD6* transgene, except plastocyanin (PETE) gene which was induced (Fig. 8a). Moreover, many genes encoding citrate cycle (TCA cycle) related enzymes were upregulated by drought, but barely by *MtPHD6* transgene (Fig. 8b). These results showed that drought stress had more extensive effect on photosynthesis and TCA cycle than *MtPHD6* transgene.

### The effect of *MtPHD6* transgene on oxidative damage under drought condition

**Fig. 9:**
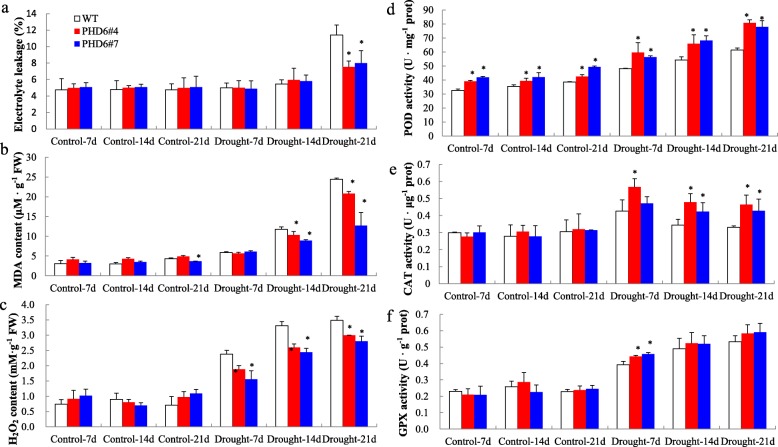
Physiological changes of *MtPHD6* transgenic lines after drought stress treatment. 12-day-old seedlings were withheld water and leaf tissues were collected at 7, 14 and 21 d after drought treatment. **a**: Electrolyte leakage (EL); **b**: Malondialdehyde (MDA) content; **c**: Hydrogen peroxide (H_2_O_2_) content; **d**: Peroxidase (POD) activity; **e**: Catalase (CAT) activity; **f**: Glutathione peroxidase (GPX) activity

Drought stress can induce membrane lipid peroxidation and ROS overproduction, resulting in changes of plasma membrane permeability. In the study, the electrolyte leakage (EL) of transgenic and WT plants showed significant increase after 21 d of drought treatment. However, transgenic plants suffered less membrane damage and exhibited significantly lower EL than WT plants at 21 d of drought stress (Fig. 9a). Malondialdehyde (MDA) content in WT plants was significantly higher than that in transgenic plants at drought stress 14 d and 21 d, respectively (Fig. 9b). Moreover, significantly lower hydrogen peroxide (H_2_O_2_) content was observed in transgenic plants compared with WT plants after drought stress (Fig. 9c).

To avoid oxidative damage, plants activate antioxidant enzymatic defense system to keep redox homeostasis. Drought treatment triggered the activities of peroxidase (POD), catalase (CAT) and glutathione peroxidase (GPX) in WT and transgenic plants when compared to control condition. The POD activity in transgenic plants was significantly higher than that in WT plants under both control and drought conditions (Fig. 9d). Furthermore, transgenic plants showed significantly higher CAT (14 d, 21 d) and GPX (7 d) activities than WT plants under drought stress (Fig. 9e, f). These results showed that transgenic plants could effectively alleviate oxidative damage when exposed to drought stress.

## Discussion

It is well known that drought stress constrains plant productivity and distribution. Through long-time evolution, plants have developed various responsive strategies to adapt to drought stress at the morphological, physiological, biochemical and molecular levels [28]. With rapidly developing analytical chemistry technologies, a large number of genes involved in drought tolerance have been excavated and functionally identified in various plant species [29, 30]. Among these genes, transcription factors play critical roles in multifarious signaling pathways that regulate plant responses to harsh environmental stresses [31–33]. So far, plenty of transcription factor families have been reported to be induced by abiotic stress, such as *WRKY*, *bZIP*, *DREB*, *MYB*/M*Y*C, *ERF*, *ZINC FINGER* and *Alfin1*-like (*AL*) families [5, 13].

Alfin1-type PHD-finger-containing proteins are involved in plant abiotic stresses apart from their roles in developmental processes [12, 34]. In this study, we found that the expression of *MtPHD6* was induced by drought stress (Figs. 1c, 2a). *MtPHD6* transgenic lines showed improved tolerance to mannitol stress during seed germination (Fig. 2). Ectopic expression of *MtPHD6* plants possessed enhanced tolerance to drought stress with significantly higher survival rates and antioxidant enzyme activities when compared to WT plants (Figs. 3b, 9). In Arabidopsis, AL proteins were found to bind to G-box elements and overexpression of *AL5* improved drought tolerance [18]. Ectopic expression of soybean *GmPHD2* increased salt tolerance in Arabidopsis probably by scavenging ROS [5]. The data in this study suggested that *MtPHD6* could function as a positive regulator in response to drought stress.

In *M. sativa*, Alfin1 binds to G-rich elements in the promoter of *MsPRP2*, a salt-inducible and root-specific gene [4, 10]. Moreover, GmPHD5 binds to the promoter and coding region of salt stress responsive genes and acts as a positive regulator in response to salt stress [16]. Through large scale transcriptome analysis, we found that genes involving in stress related GO terms were enriched by drought stress and *MtPHD6* transgene (Fig. 5, Additional file 8: Figure S2a), including ABA signaling pathway (Fig. 7d). In rice (*O. sativa*), five *OsPHD* genes were significantly induced while two inhibited by ABA [35]. In soybean, expression of *GmPHD 1–6* were induced after ABA and drought treatment, and *GmPHD2* transgene upregulated expression of ABA responsive bZIP transcription factor *ABI5* [5]. In this study, *MtPHD6* transgene regulated different subset of ABA responsive genes like *PYL4–6* (Fig. 7d). As the key hormone during plant responses to drought stress, ABA can activate PYR/PYL-PP2C-SnRK2-ABF signaling pathway, which plays vital roles in plant drought tolerance. ABA pathways might function as the upstream signaling in *MtPHD6* transgenic Arabidopsis in response to drought stress.

Similar to RING finger and LIM domain, the PHD finger is characterized as a conserved Cys_4_HisCys_3_-type zinc finger type. In Arabidopsis, *ZINC FINGER* and *WRKY* type transcription factors were identified as *AL5*-regulated genes by ChIP-Seq [18]. Indeed, several zinc finger transcription factors were modulated by *MtPHD6* transgene and part of these genes were also induced by drought stress or ABA treatment (Fig. 7a). Arabidopsis zinc finger proteins *ZAT6* and *ZAT18* were transcriptionally induced by drought, salt, cold and heat stresses [26, 27]. Overexpression of Arabidopsis *ZAT6*, *ZAT18* and pepper *ZFP1* increased the expression level of stress induced genes and ABA signaling pathway related genes, resulting in enhanced drought tolerance [26, 27, 36]. *AtZAT7* overexpression inhibited plant growth and enhanced salt tolerance of transgenic Arabidopsis [37]. Under stress condition, *AtAZF1* and *AtAZF2* had a negative effect on the expression of auxin-inducible and ABA repressive genes [31]. Moreover, a Cys_2_His_2_-type zinc finger transcription factor from *Glyycine soja*, *GsZFP1*, could be induced by abiotic stresses and ABA treatment. Ectopic expression of *GsZFP1* in Arabidopsis activated the expression of ABA biosynthesis related genes and increased tolerance to drought stress [38]. These data showed that zinc finger transcription factors can function as positive regulators in response to drought stress. PHD domain transcription factors including Alfin1 encode novel members of zinc finger family proteins [5, 24]. *MtPHD6* transgene upregulated the expressions of *RZPF34*, *ZAT7*, *STZ*/*ZAT10*, *SZF1*, *ZF1*, and several *C3HC4* zinc finger transcription factors in Arabidopsis, which were induced after ABA treatment (Fig. 7a). These results indicated that several zinc finger proteins might function as ABA downstream regulators and play important roles in drought tolerance of *MtPHD6* transgenic plants.

*AP2/EREBP* and *WRKY* were shown to play key roles in plant abiotic stress responses. *AP2/EREBP* family is a large group of plant-specific transcription factors, and has four major subfamilies, including the *AP2*, *DREB*, *ERF* and *RAV* [39]. Totally 123 putative *AP2/ERF* genes were identified in *M. truncatula* genome. Further analysis showed that cold stress induced a cluster of *DREB* subfamily genes on chromosome 6 indicating the contribution of these genes in response to cold stress [40]. Overexpression of tomato *DREB2* improved salt tolerance in transgenic tomato and Arabidopsis through activation of proline and polyamines biosynthesis and increase of K^+^/Na^+^ ratio [41]. Vitis *CBF1* and *CBF4* transgenic Arabidopsis showed improved cold and drought stress tolerances. Different subsets of stress responsive genes were modulated by *VrCBF1* and *VrCBF4* transgene, indicating that Vitis *CBFs* have different regulons [42]. Additionally, *WRKY* family also plays a crucial role in regulating plant responses to abiotic stresses, including drought, cold, salt and osmotic stresses [43]. Ectopic expression of sunflower *HaWRKY76* increased drought and flood stress tolerances of transgenic Arabidopsis plants [44]. Wheat *TaWRKY1* and *TaWRKY33* conferred drought and heat stress tolerance in Arabidopsis [45]. Mutation of Arabidopsis *WRKY* transcription factor *ABO3* resulted in decreased expression of *ABF2/AREB1*, *RD29A* and *COR47*, indicating the important role of *WRKY* in plant stress responses [46]. Ectopic expression of *MtPHD6* increased the expression of *AP2/EREBP* and *WRKY* transcription factors in Arabidopsis (Fig. 6, Additional file 9: Figure S3). Therefore, *MtPHD6* transgene modulated *AP2*/*EREBP* and *WRKY* transcription factors might contribute to enhanced plant drought tolerance.

## Conclusions

**Fig. 10:**
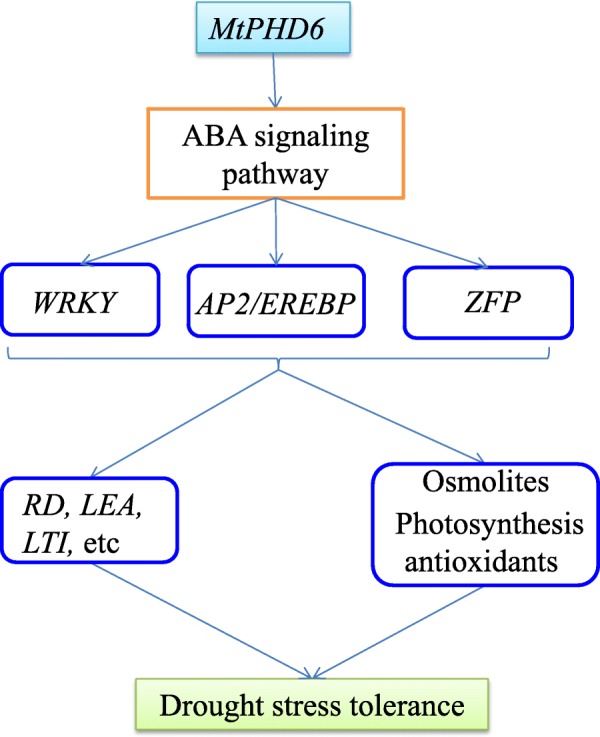
Model depicting roles of *MtPHD6* in response to drought stress

Taken together, *MtPHD6* transgene might activate stress responsive *ZINC FINGER*, *AP2*/*EREBP* and *WRKY* transcription factors through ABA dependent and independent pathways. These transcription factors interact with stress related proteins, regulate other stress responsive genes including *NAC*, *CML*, *RD*, *LEA*, *LTI* and *MPK*, and/or modulate accumulation of osmolites, photosynthesis, and ROS metabolism, resulting in improved plant drought stress tolerance (Fig. 10).

## Methods

### Plant materials and growth conditions

In this study, *M. truncatula* Jemalong A17 and *A. thaliana* Columbia-0 were used and identified by Wuhan Botanic Garden. Seeds of *M. truncatula* Jemalong A17 were provided by Dr. Haiqing Wang from Chinese Academy of Sciences and seeds of *A. thaliana* Columbia-0 were self-propagated in the lab.

After surface-sterilization in 2% NaClO solution for 5 min, Arabidopsis seeds were stratified in deionized water at 4 °C for 3 d in darkness. Then the seeds were sown on full-strength Murashige and Skoog (MS) medium plates (pH 5.7), and were incubated in a growth chamber at 22 °C with 120 μmol m^− 2^ s^− 1^ irradiance, 60% relative humidity, and a light/ dark cycle of 16/8 h. After growth for 7 d, the seedlings, except those used in germination tests, were transferred into soil-filled containers and irrigated with nutrient solution every 3 days.

### Cloning of *MtPHD6* and gene transformation

The full-length coding sequence of *MtPHD6* from *M. truncatula* was isolated by PCR amplification using MtPHD6-F and MtPHD6-R primers with *KpnI* and B*amHI* restriction sites, respectively (Additional file 2: Table S2). Then PCR products were purified and inserted into pCAMBIA1301S vectors to construct recombinant plasmids. The transformed *A. thaliana* plants were generated through introduction of *Agrobacterium tumefaciens* strain GV3101 containing the constructs using the floral lip method [47]. The positive transgenic plants were identified on MS medium with 25 μg ml^− 1^ hygromycin under the same growth condition. T3 homozygous lines were confirmed by PCR analyses and used for following experiments.

### Germination tests and drought treatment

The sensitivity of *MtPHD6*-overexpressing seeds to mannitol was analyzed on MS medium plates at the germination stage. After vernalization, WT and transgenic seeds were sown on MS medium plates supplemented with or without 300 mM mannitol, respectively. It was considered as germinated seeds when radicles fully penetrated the seed coat. Germinated seeds were counted daily for 7 d.

For the 12-day-old soil-grown seedlings, water was withheld for 23 d before rehydration. Leaf samples under control and stress conditions were harvested at 7 d, 14 d and 21 d after drought treatment, respectively. The survival rate was calculated at 2 d after re-watering.

### Analysis of water loss and leaf water content

To analyze water loss under control condition, detached leaves from 21-day-old plants were weighted at 1 h intervals for up to 8 h. The leaf samples at different intervals after drought stress were used for measurement of leaf water content (LWC). Fresh weight (FW) of leaf samples was immediately quantified, and dry weight (DW) was measured after 16 h at 80 °C oven. LWC (%) calculation was made as follows: (FW-DW)/FW × 100 [26].

### Measurement of EL and MDA

EL was measured as described [48]. Briefly, detached leaves were placed in a tube with 15 mL deionized water on a shaker for 6 h. The C_i_ of leaf samples was determined before boiling for 20 min. After boiling and cooling to room temperature, the C_max_ was measured and then the percentage of EL was computed based on the formula of (C_i_/C_max_) × 100.

MDA determination was performed as described [27]. Briefly, 0.3 g leaf samples were ground with liquid nitrogen and 5% trichloroacetic acid (TCA; w/v) was added and mixed with fine leaf powder. Before adding 2 mL of 0.67% thiobarbituric acid (TBA, w/v), the supernatants were centrifuged at 12,000 g for 10 min. The solutions were mixed and boiled for 30 min. After centrifugation and cooling to room temperature, the concentration of MDA was computed according to the absorbance at 450, 532 and 600 nm, respectively.

### Quantification of H_2_O_2_ content and antioxidant enzyme activities

For the assessment of H_2_O_2_ content and antioxidant enzyme activities, the supernatant from fresh leaves was prepared according to Quan et al. [49] and used for the following measurement.

The content of H_2_O_2_ was analyzed using H_2_O_2_ Assay Kit (A064–1-1, Nanjing Jiancheng Bioengineering Institute, China) based on the manufacturer’s instructions. The measurement of POD, CAT and GPX activities was performed with Plant POD Assay Kit (A084–3-1, Nanjing Jiancheng Bioengineering Institute, China), CAT Assay Kit (A007–1-1, Nanjing Jiancheng Bioengineering Institute, China) and GPX Assay Kit (A005–1-2, Nanjing Jiancheng Bioengineering Institute, China), respectively, as the introduction described.

### RNA isolation and quantitative real-time PCR

Leaf samples of WT and *MtPHD6* transgenic lines after 7 d of drought treatment were collected for RNA isolation. Using Trizol reagent (Invitrogen, Carlsbad, CA, USA), total RNA was extracted and then treated by DNase I (Promega, Madison, WI, USA) to remove the contamination of genomic DNA [48, 49]. RNA concentration and purity were measured by the NanoPhotometer® spectrophotometer (IMPLEN, CA, USA). The integrity of RNA was assessed with 1.5% (w/v) agarose gel electrophoresis. Totally 2 μg of above RNA was reverse-transcribed into cDNA using reverse transcriptase (TOYOBO, Ohtsu, Janpan) according to the manufacturer’s instructions.

qRT-PCR was executed by a CFX 96 Real Time System (Bio-Rad, California, USA) with SYBR green fluorescence. The expression levels of genes were standardized with ubiquitin 10 (AtUBQ10, AT4G05320) and calculated by the 2^-ΔΔCT^ method [50]. Gene-specific primer sequences used for qRT-PCR are listed in Additional file 2: Table S2. The experiment had three technical and biological replicates.

### Transcriptomic analysis

RNA-seq analysis was performed by the Novogene Corporation (Beijing, China) as described [27]. Totally 3 μg RNA for each sample was used for preparation of sequencing libraries using NEBNext® UltraTM RNA Library Prep Kit for Illumina®. Index codes were added to attribute sequences and the clustering of index-coded samples was performed on a cBot Cluster Generation System (Illumina). Library preparations were then sequenced on an Illumina Hiseq platform and paired-end reads with 125 bp/150 bp were generated. After removing reads containing poly-N, adapter and low quality reads from raw data, clean data (clean reads) were generated. Paired-end clean reads were aligned to the Arabidopsis genome using Hisat2 v2.0.5. The read numbers mapped to each gene were counted using featureCounts v1.5.0-p3, and the FPKM of each gene was computed according to the length of the gene and read counts mapped to this gene. Differential expression analysis of each treatment versus control was investigated with the DESeq2 R package (1.16.1). There were 12 samples with three biological replicates for each sample. DEGs were selected with a fold change > 2 and *P*-value < 0.05. The RNA-seq data have been submitted to NCBI Gene Expression Omnibus (GEO) with the accession number GSE134945.

### Hierarchical cluster analysis

Cluster 3.0 program (http://bonsai.hgc.jp/~mdehoon/software/cluster/software.htm) was used for hierarchical cluster analysis with uncentered matrix and complete linkage method. The resulting tree figures were displayed with Java Treeview software (http://jtreeview.sourceforge.net/) [51].

### GO term and pathway enrichment analysis

All DEGs from RNA-seq data were annotated using the Classification SuperViewer Tool (http://bar.utoronto.ca/ntools/cgi-bin/ntools_classification_superviewer.cgi) for GO term and pathway enrichment analysis [52]. GO and MapMan were respectively chosen as classification source. The normalized frequency (NF) of each functional category was computed according to the following formula: NF = sample frequency in each sample/background frequency in Arabidopsis genome.

### Transcriptional network analysis

Interaction networks of DEGs were analyzed using STRING database (https://string-db.org/) with high confidence (score > 0.7) [53]. Tab separated values from STRING were then analyzed with Cytoscape software (http://www.cytoscape.org/).

### Statistical analysis

All experiments were repeated three times independently. Leaf samples used in each experiment were collected from 3 containers with at least 15 plants of each container. The results presented were the mean ± SD. The significant differences relative to WT were showed by asterisks above the columns of figures at *P* ≤ 0.05 level using Student’s t-test.

## Supplementary information

**Additional file 1:** **Table S1.** Genbank accession numbers of AL/PHD proteins used for phylogenetic analysis

**Additional file 2:** **Table S2.** Primer sequences used for gene cloning and qRT-PCR experiments

**Additional file 3:** **Table S3.** Spreadsheet of genes changed by drought stress or *MtPHD6* transgene.

**Additional file 4:** **Table S4.** Spreadsheet of genes commonly regulated by *MtPHD6* transgene and drought stress

**Additional file 5:** **Table S5.** Spreadsheet of genes for MapMAN pathway analysis.

**Additional file 6:** **Table S6.** Spreadsheet of specific genes including ABA pathway related genes, zinc finger, AP2/ EREBP and WRKY transcription factors.

**Additional file 7:** **Figure S1.** Correlation expression analysis of selected genes by RNA-seq and qRT-PCR. Totally 11 genes co-regulated by *MtPHD6* transgene and drought treatment were selected for qRT-PCR analysis.

**Additional file 8:** **Figure S2.** GO term enrichment analysis of genes changed by drought or *MtPHD6* transgene in Arabidopsis.

**Additional file 9:** **Figure S3.** Cluster and network analyses of AP2/EREBP and WRKY transcription factors. For cluster analysis, the list of genes were analyzed using Cluster 3.0 and the resulting tree figure was shown by Java Treeview. Network analysis was performed using STRING (https://string-db.org) and Cytoscape 3.7.1. a: Cluster analysis of AP2/EREBP transcription factors affected by *MtPHD6* transgene, drought and ABA. b and c: Networks of AP2/EREBP transcription factors modulated by drought stress (green node and red edge) and *MtPHD6* transgene (red node and green edge), respectively. d: Cluster analysis of WRKY transcription factors affected by *MtPHD6* transgene, drought and ABA. e and f: Networks of WRKY transcription factors modulated by drought stress (green node and red edge) and *MtPHD6* transgene (red node and green edge), respectively. The original data were presented in Additional file 6: Table S6. ABA treatment expression data were downloaded from BAR Expression Browser (http://bar.utoronto.ca/affydb/cgi-bin/affy_db_exprss_browser_in.cgi). The grey color in cluster analysis indicates the genes are missing in BAR database.

## Data Availability

Sequence data from this article can be found in GEO data library (https://www.ncbi.nlm.nih.gov/geo/) under the accession number GSE134945. DEGs analysis is included in Additional files 3, 4, 5, 6, 7, 8 and 9.

## References

[1] Schindler U, Beckmann H, Cashmore AR (1993). HAT3.1, a novel *Arabidopsis* homeodomain protein containing a conserved cysteine-rich region. Plant J.

[2] Aasland R, Gibson TJ, Stewart AF (1995). The PHD finger: implications for chromatinmediated transcriptional regulation. Trends Biochem Sci.

[3] Bienz M (2006). The PHD finger, a nuclear protein-interaction domain. Trends Biochem Sci.

[4] Bastola DR, Pethe VV, Winicov I (1998). Alfin1, a novel zinc-finger protein in alfalfa roots that binds to promoter elements in the salt-inducible *MsPRP2* gene. Plant Mol Biol.

[5] Wei W, Huang J, Hao YJ, Zou HF, Wang HW, Zhao JY (2009). Soybean GmPHD-type transcription regulators improve stress tolerance in transgenic Arabidopsis plants. PLoS One.

[6] Chandrika NNP, Sundaravelpandian K, Schmidt W (2013). A PHD in histone language: on the role of histone methylation in plant responses to phosphate deficiency. Plant Signal Behav.

[7] Kayum MA, Park JI, Ahmed NU, Jung HJ, Saha G, Kang JG (2015). Characterization and stress-induced expression analysis of Alfin-like transcription factors in *Brassica rapa*. Mol Gen Genomics.

[8] Sanchez MDLP, Gutierrez C (2009). *Arabidopsis* ORC1 is a PHD containing H3K4me3 effector that regulates transcription. Proc Natl Acad Sci U S A.

[9] Sung S, Schmitz RJ, Amasino RM (2006). A PHD finger protein involved in both the vernalization and photoperiod pathways in *Arabidopsis*. Gene Develop.

[10] Winicov I, Valliyodan B, Xue L, Hoober JK (2004). The *MsPRP2* promoter enables strong heterologous gene expression in a root-specific manner and is enhanced by overexpression of *Alfin 1*. Planta.

[11] Xiong Y, Liu T, Tian C, Sun S, Li J, Chen M (2005). Transcription factors in rice: a genome-wide comparative analysis between monocots and eudicots. Plant Mol Biol.

[12] Zhou W, Wu J, Zheng Q, Jiang Y, Zhang M, Zhu S (2017). Genome-wide identification and comparative analysis of Alfin-like transcription factors in maize. Genes Genom.

[13] Tao JJ, Wei W, Pan WJ, Lu L, Li QT, Ma JB (2018). An *Alfin-like* gene from *Atriplex hortensis* enhances salt and drought tolerance and abscisic acid response in transgenic *Arabidopsis*. Sci Rep.

[14] Qu L, Zhu Y (2006). Transcription factor families in *Arabidopsis*: major progress and outstanding issues for future research. Curr Opin Plant Biol.

[15] Song Y, Gao J, Yang F, Kua CS, Liu J, Cannon CH (2013). Molecular evolutionary analysis of the Alfin-like protein family in *Arabidopsis lyrata*, *Arabidopsis thaliana*, and *Thellungiella halophila*. PLoS One.

[16] Wu T, Pi EX, Tsai SN, Lam HM, Sun SM, Kwan YW (2011). GmPHD5 acts as an important regulator for crosstalk between histone H3K4 di-methylation and H3K14 acetylation in response to salinity stress in soybean. BMC Plant Biol.

[17] Lee WY, Lee D, Chung WI, Kwon CS (2009). *Arabidopsis* ING and Alfin1-like protein families localize to the nucleus and bind to H3K4me3/2 via plant homeodomain fingers. Plant J.

[18] Wei W, Zhang YQ, Tao JJ, Chen HW, Li QT, Zhang WK (2015). The Alfin-like homeodomain finger protein AL5 suppresses multiple negative factors to confer abiotic stress tolerance in *Arabidopsis*. Plant J.

[19] Zhang JY, Broeckling CD, Blancaflor EB, Sledge MK, Summer LW, Wang ZY (2005). Overexpression of *WXP1*, a putative *Medicago truncatula* AP2 domain-containing transcription factor gene, increases cuticular wax accumulation and enhances drought tolerance in transgenic alfalfa (*Medicago sativa*). Plant J.

[20] May GD, Hopkins A, Wang ZY, Mian R, Sledge M, Barker RE (2004). From models to crops: integrated medicago genomics for alfalfa improvement. Molecular breeding of forage and turf. Developments in plant breeding.

[21] Wang ZY, Hopkins A, Mian R (2001). Forage and turf grass biotechnology. Crit Rev Plant Sci.

[22] Yu Y, Bi C, Wang Q, Ni Z (2019). Overexpression of *TaSIM* provides increased drought stress tolerance in transgenic *Arabidopsis*. Biochem Bioph Res Co.

[23] Gou J, Debnath S, Sun L, Flanagan A, Tang Y, Jiang Q (2018). From model to crop: functional characterization of *SPL8* in *M. truncatula* led to genetic improvement of biomass yield and abiotic stress tolerance in alfalfa. Plant Biotech J.

[24] Winicov I, Bastola DR (1999). Transgenic overexpression of the transcription factor *Alfin 1* enhances expression of the endogenous *MsPRP2* gene in alfalfa and improves salinity tolerance of the plants. Plant Physiol.

[25] Medina J, Catalá R, Salinas J (2011). The *CBFs*: three Arabidopsis transcription factors to cold acclimate. Plant Sci.

[26] Shi H, Wang X, Ye T, Chen F, Deng J, Yang P (2014). The cysteine2/histidine2-type transcription factor *ZINC FINGER OF ARABIDOPSIS THALIANA6* modulates biotic and abiotic stress responses by activating salicylic acid-related genes and *C-REPEAT-BINDING FACTOR* genes in *Arabidopsis*. Plant Physiol.

[27] Yin M, Wang Y, Zhang L, Li J, Quan W, Yang L (2017). The *Arabidopsis* Cys2/His2 zinc-finger transcription factor *ZAT18* is a positive regulator of plant tolerance to drought stress. J Exp Bot.

[28] Zhu JK (2002). Salt and drought stress signal transduction in plants. Annu Rev Plant Biol.

[29] Lei Y, Xu Y, Hettenhausen C, Lu C, Shen G, Zhang C (2018). Comparative analysis of alfalfa (*Medicago sativa* L.) leaf transcriptomes reveals genotype-specific salt tolerance mechanisms. BMC Plant Biol.

[30] Zhao Y, Chan Z, Gao J, Xing L, Cao M, Yu C (2016). The ABA receptor PYL9 promotes drought resistance and leaf senescence. Proc Natl Acad Sci U S A.

[31] Kodaira KS, Qin F, Tran LSP, Maruyama K, Kidokoro S, Fujita Y (2011). Arabidopsis Cys2/His2 zinc-finger proteins AZF1 and AZF2 negatively regulate abscisic acid repressive and auxin-inducible genes under abiotic stress conditions. Plant Physiol.

[32] Ma Q, Dai X, Xu Y, Guo J, Liu Y, Chen N (2009). Enhanced tolerance to chilling stress in *OsMYB3R-2* transgenic rice is mediated by alteration in cell cycle and ectopic expression of stress genes. Plant Physiol.

[33] Xie Y, Chen P, Yan Y, Bao C, Li X, Wang L (2018). An atypical R2R3 *MYB* transcription factor increases cold hardiness by *CBF*-dependent and *CBF*-independent pathways in apple. New Phytol.

[34] Mouriz A, López-González L, Jarillo JA, Piñeiro M (2015). PHDs govern plant development. Plant Signal Behav.

[35] Sun M, Jia B, Yang J, Cui N, Zhu Y, Sun X (2017). Genome-wide identification of the PHD-finger family genes and their responses to environmental stresses in *Oryza sativa* L. Int J Mol Sci.

[36] Kim SH, Hong JK, Lee SC, Sohn KH, Jung HW, Hwang BK (2004). *CAZFP1*, Cys_2_/His_2_-type zinc-finger transcription factor gene functions as a pathogen-induced early-defense gene in *Capsicum annuum*. Plant Mol Biol.

[37] Ciftci-Yilmaz S, Morsy MR, Song L, Coutu A, Krizek BA, Lewis MW (2007). The EAR-motif of the Cys2/His2-type zinc finger protein Zat7 plays a key role in the defense response of *Arabidopsis* to salinity stress. J Biol Chem.

[38] Luo X, Bai X, Zhu D, Li Y, Ji W, Cai H (2012). *GsZFP1*, a new Cys_2_/His_2_-type zinc-finger protein, is a positive regulator of plant tolerance to cold and drought stress. Planta.

[39] Mizoi J, Shinozaki K, Yamaguchi-Shinozaki K (2012). AP2/ERF family transcription factors in plant abiotic stress responses. BBA-Gene Regul Mech.

[40] Shu Y, Liu Y, Zhang J, Song L, Guo C (2016). Genome-wide analysis of the AP2/ERF superfamily genes and their responses to abiotic stress in *Medicago truncatula*. Front Plant Sci.

[41] Hichri I, Muhovski Y, Clippe A, Žižková E, Dobrev PI, Motyka V (2016). *SlDREB2*, a tomato dehydration-responsive element-binding 2 transcription factor, mediates salt stress tolerance in tomato and Arabidopsis. Plant Cell Environ.

[42] Siddiqua M, Nassuth A (2011). Vitis *CBF1* and Vitis *CBF4* differ in their effect on Arabidopsis abiotic stress tolerance, development and gene expression. Plant Cell Environ.

[43] Tripathi P, Rabara RC, Rushton PJ (2014). A systems biology perspective on the role of WRKY transcription factors in drought responses in plants. Planta.

[44] Raineri J, Ribichich KF, Chan RL (2015). The sunflower transcription factor *HaWRKY76* confers drought and flood tolerance to *Arabidopsis thaliana* plants without yield penalty. Plant Cell Rep.

[45] He GH, Xu JY, Wang YX, Guo W, Zhang X (2006). Drought-responsive WRKY transcription factor genes *TaWRKY1* and *TaWRKY33* from wheat confer drought and/or heat resistance in Arabidopsis. BMC Plant Biol.

[46] Ren X, Chen Z, Liu Y, Zhang H, Zhang M, Liu Q (2010). *ABO3*, a WRKY transcription factor, mediates plant responses to abscisic acid and drought tolerance in Arabidopsis. Plant J.

[47] Clough SJ, Bent AF (1998). Floral dip: a simplified method for agrobacterium-mediated transformation of Arabidopsis thaliana. Plant J.

[48] Quan W, Liu X, Wang H, Chan Z (2016). Comparative physiological and transcriptional analyses of two contrasting drought tolerant alfalfa varieties. Front Plant Sci.

[49] Quan W, Liu X, Wang H, Chan Z (2016). Physiological and transcriptional responses of contrasting alfalfa (*Medicago sativa* L.) varieties to salt stress. Plant cell tissue organ. Cult.

[50] Livak KJ, Schmittgen TD (2001). Analysis of relative gene expression data using real-time itative PCR and the 2^− ΔΔCT^ method. Methods.

[51] Chan Z, Bigelow PJ, Loescher W, Grumet R (2012). Comparison of salt stress resistance genes in transgenic *Arabidopsis thaliana* indicates that extent of transcriptomic change may not predict secondary phenotypic or fitness effects. Plant Biotech J.

[52] Provart NJ, Zhu T (2003). A browser-based functional classification superviewer for *Arabidopsis* genomics. Curr Comput Mol Biol.

[53] Szklarczyk D, Morris JH, Cook H, Kuhn M, Wyder S, Simonovic M (2017). The STRING database in 2017: quality-controlled protein-protein association networks, made broadly accessible. Nucleic Acids Res.

