# Supplementary material for: Ectopic expression of Medicago truncatula homeodomain finger protein, MtPHD6, enhances drought tolerance in Arabidopsis
Source: BMC Genomics. 2019 Dec 16;20:982. doi: 10.1186/s12864-019-6350-5 (PMC6916436; doi:10.1186/s12864-019-6350-5)
Supplement: Supplementary file 1 — Additional file 1: Table S1. Genbank accession numbers of AL/PHD proteins used for phylogenetic analysis [file 12864_2019_6350_MOESM1_ESM.pptx]

## Slide 1
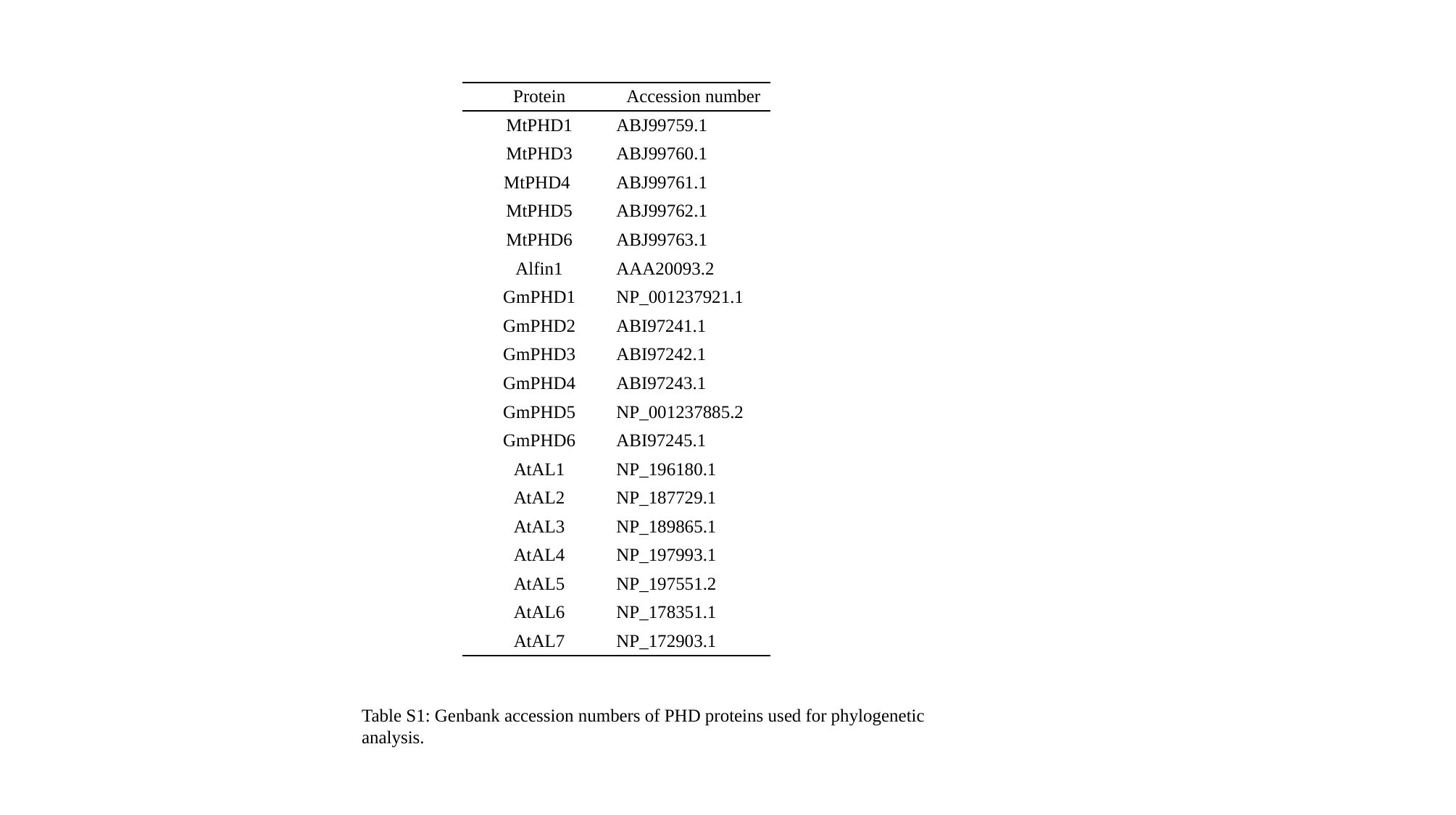

| Protein | Accession number |
| --- | --- |
| MtPHD1 | ABJ99759.1 |
| MtPHD3 | ABJ99760.1 |
| MtPHD4 | ABJ99761.1 |
| MtPHD5 | ABJ99762.1 |
| MtPHD6 | ABJ99763.1 |
| Alfin1 | AAA20093.2 |
| GmPHD1 | NP\_001237921.1 |
| GmPHD2 | ABI97241.1 |
| GmPHD3 | ABI97242.1 |
| GmPHD4 | ABI97243.1 |
| GmPHD5 | NP\_001237885.2 |
| GmPHD6 | ABI97245.1 |
| AtAL1 | NP\_196180.1 |
| AtAL2 | NP\_187729.1 |
| AtAL3 | NP\_189865.1 |
| AtAL4 | NP\_197993.1 |
| AtAL5 | NP\_197551.2 |
| AtAL6 | NP\_178351.1 |
| AtAL7 | NP\_172903.1 |
Table S1: Genbank accession numbers of PHD proteins used for phylogenetic analysis.
