# Supplementary material for: Ectopic expression of Medicago truncatula homeodomain finger protein, MtPHD6, enhances drought tolerance in Arabidopsis
Source: BMC Genomics. 2019 Dec 16;20:982. doi: 10.1186/s12864-019-6350-5 (PMC6916436; doi:10.1186/s12864-019-6350-5)
Supplement: Supplementary file 2 — Additional file 2: Table S2. Primer sequences used for gene cloning and qRT-PCR experiments [file 12864_2019_6350_MOESM2_ESM.pptx]

## Slide 1
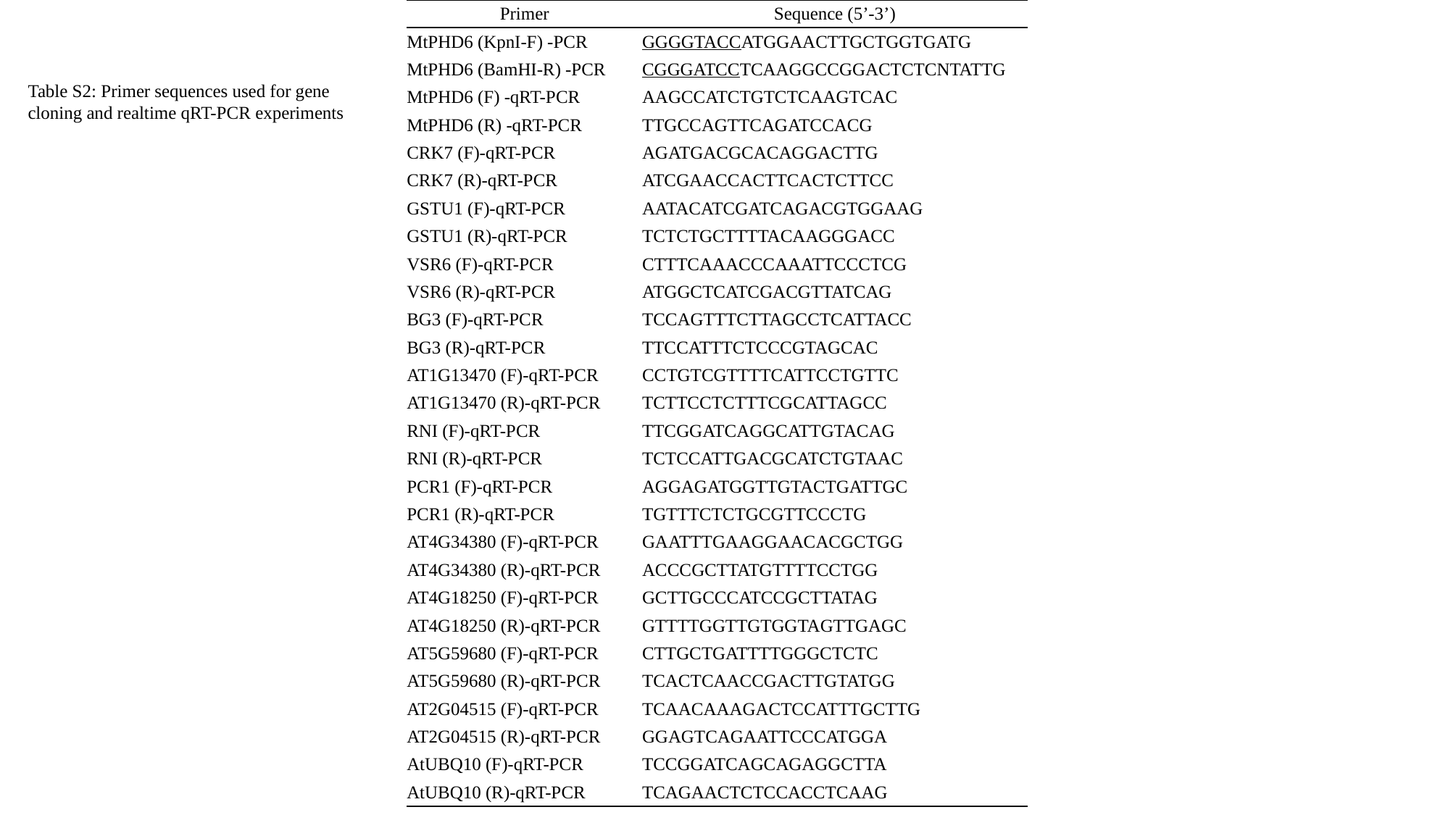

| Primer | Sequence (5’-3’) |
| --- | --- |
| MtPHD6 (KpnI-F) -PCR | GGGGTACCATGGAACTTGCTGGTGATG |
| MtPHD6 (BamHI-R) -PCR | CGGGATCCTCAAGGCCGGACTCTCNTATTG |
| MtPHD6 (F) -qRT-PCR | AAGCCATCTGTCTCAAGTCAC |
| MtPHD6 (R) -qRT-PCR | TTGCCAGTTCAGATCCACG |
| CRK7 (F)-qRT-PCR | AGATGACGCACAGGACTTG |
| CRK7 (R)-qRT-PCR | ATCGAACCACTTCACTCTTCC |
| GSTU1 (F)-qRT-PCR | AATACATCGATCAGACGTGGAAG |
| GSTU1 (R)-qRT-PCR | TCTCTGCTTTTACAAGGGACC |
| VSR6 (F)-qRT-PCR | CTTTCAAACCCAAATTCCCTCG |
| VSR6 (R)-qRT-PCR | ATGGCTCATCGACGTTATCAG |
| BG3 (F)-qRT-PCR | TCCAGTTTCTTAGCCTCATTACC |
| BG3 (R)-qRT-PCR | TTCCATTTCTCCCGTAGCAC |
| AT1G13470 (F)-qRT-PCR | CCTGTCGTTTTCATTCCTGTTC |
| AT1G13470 (R)-qRT-PCR | TCTTCCTCTTTCGCATTAGCC |
| RNI (F)-qRT-PCR | TTCGGATCAGGCATTGTACAG |
| RNI (R)-qRT-PCR | TCTCCATTGACGCATCTGTAAC |
| PCR1 (F)-qRT-PCR | AGGAGATGGTTGTACTGATTGC |
| PCR1 (R)-qRT-PCR | TGTTTCTCTGCGTTCCCTG |
| AT4G34380 (F)-qRT-PCR | GAATTTGAAGGAACACGCTGG |
| AT4G34380 (R)-qRT-PCR | ACCCGCTTATGTTTTCCTGG |
| AT4G18250 (F)-qRT-PCR | GCTTGCCCATCCGCTTATAG |
| AT4G18250 (R)-qRT-PCR | GTTTTGGTTGTGGTAGTTGAGC |
| AT5G59680 (F)-qRT-PCR | CTTGCTGATTTTGGGCTCTC |
| AT5G59680 (R)-qRT-PCR | TCACTCAACCGACTTGTATGG |
| AT2G04515 (F)-qRT-PCR | TCAACAAAGACTCCATTTGCTTG |
| AT2G04515 (R)-qRT-PCR | GGAGTCAGAATTCCCATGGA |
| AtUBQ10 (F)-qRT-PCR | TCCGGATCAGCAGAGGCTTA |
| AtUBQ10 (R)-qRT-PCR | TCAGAACTCTCCACCTCAAG |
Table S2: Primer sequences used for gene cloning and realtime qRT-PCR experiments
