# Supplementary material for: Ectopic expression of Medicago truncatula homeodomain finger protein, MtPHD6, enhances drought tolerance in Arabidopsis
Source: BMC Genomics. 2019 Dec 16;20:982. doi: 10.1186/s12864-019-6350-5 (PMC6916436; doi:10.1186/s12864-019-6350-5)
Supplement: Supplementary file 7 — Additional file 7: Figure S1. Correlation expression analysis of selected genes by RNA-seq and qRT-PCR. Totally 11 genes co-regulated by MtPHD6 transgene and drought treatment were selected for qRT-PCR analysis. [file 12864_2019_6350_MOESM7_ESM.pptx]

## Slide 1
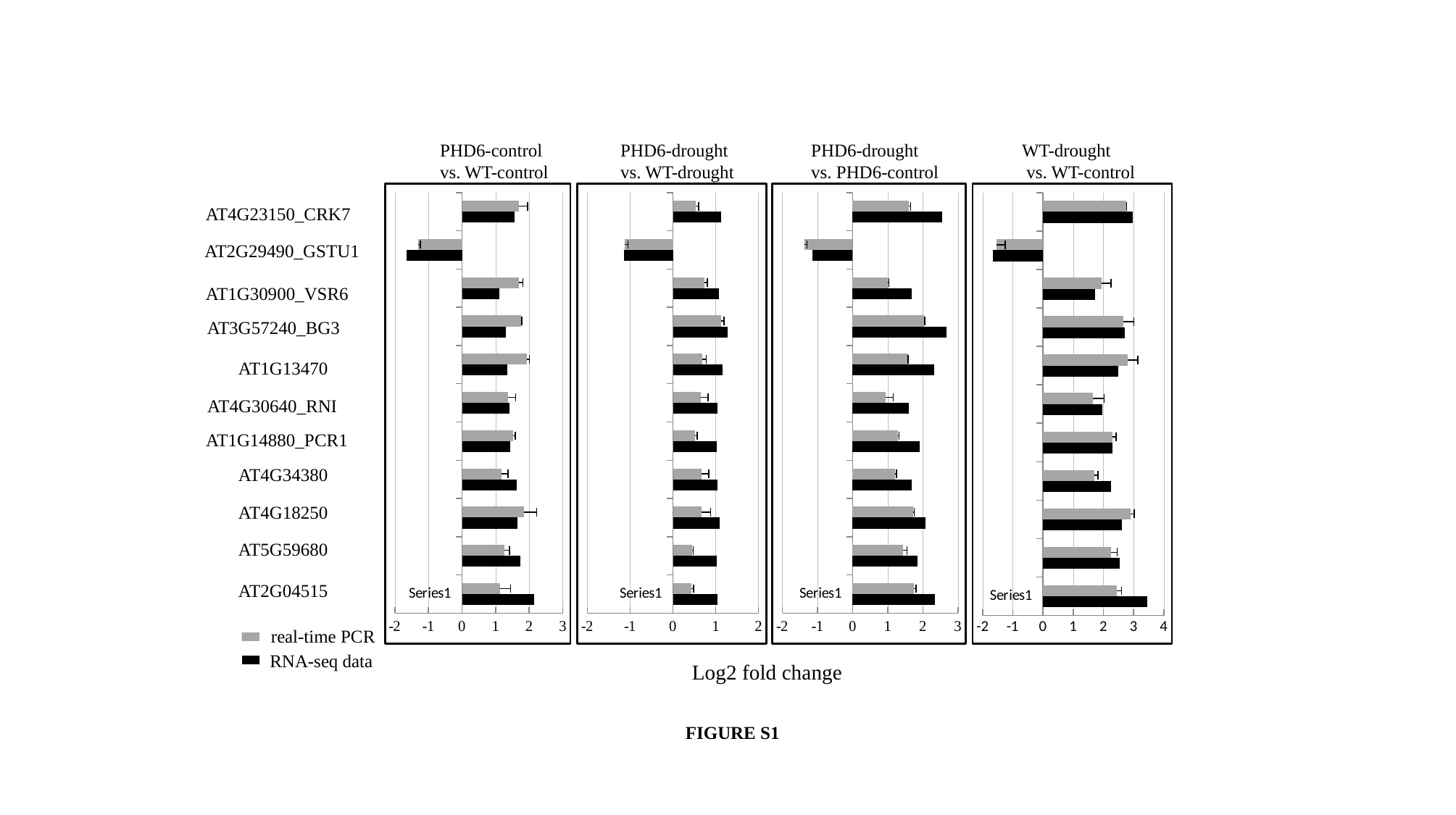

PHD6-control vs. WT-control
PHD6-drought
vs. WT-drought
PHD6-drought
vs. PHD6-control
WT-drought
 vs. WT-control
### Chart
| Category | RNA-seq data | real-time PCR |
|---|---|---|
| | 2.1547054899852367 | 1.138235092163086 |
| | 1.73074102063973 | 1.2602965037028007 |
| | 1.6493400669978904 | 1.8524869283040475 |
| | 1.63535385158063 | 1.1709060668945321 |
| | 1.4263363159919678 | 1.5139369964599452 |
| | 1.4172919628481098 | 1.361886342366537 |
| | 1.33927382561114 | 1.9235267639160165 |
| | 1.31038440775135 | 1.7510058085123699 |
| | 1.10721761110148 | 1.693761825561524 |
| | -1.6610463477940098 | -1.3075962066650286 |
| | 1.563476548603 | 1.6840820312500244 |
### Chart
| Category | RNA-seq data | real-time PCR |
|---|---|---|
| | 1.0356767556379245 | 0.4326903025309239 |
| | 1.02360516834102 | 0.4391568501790359 |
| | 1.08856802599935 | 0.668031056722011 |
| | 1.0398215751938698 | 0.673838297526049 |
| | 1.0271679398771 | 0.5074272155761727 |
| | 1.04338682060763 | 0.6587034861246843 |
| | 1.1589098158153999 | 0.6871808369954436 |
| | 1.27264858907509 | 1.1211477915445967 |
| | 1.06713800945083 | 0.7423464457194004 |
| | -1.1366650782160101 | -1.1255970001220699 |
| | 1.1183755381628604 | 0.5295931498209521 |
### Chart
| Category | RNA-seq data | real-time PCR |
|---|---|---|
| | 2.34086486523613 | 1.7458184560139978 |
| | 1.84817774610734 | 1.4306424458821598 |
| | 2.077121885587786 | 1.7221043904622289 |
| | 1.6734468452146398 | 1.1974493662516281 |
| | 1.90086894215082 | 1.29405148824056 |
| | 1.6032324908429698 | 0.9437745412190801 |
| | 2.3198516633744033 | 1.5669937133789058 |
| | 2.681439268680609 | 2.033501942952515 |
| | 1.6799697164510499 | 0.9973551432291661 |
| | -1.14156708432868 | -1.3703695933024078 |
| | 2.5401130792570012 | 1.607373555501302 |
### Chart
| Category | RNA-seq data | real-time PCR |
|---|---|---|
| | 3.4507129896814077 | 2.45136324564616 |
| | 2.5488198361001877 | 2.2517820994059257 |
| | 2.6158261608158377 | 2.9065602620442714 |
| | 2.26211069176686 | 1.6945171356201327 |
| | 2.294077008016586 | 2.300561269124349 |
| | 1.9691544191997 | 1.6469573974609375 |
| | 2.4900193806702777 | 2.8033396402994812 |
| | 2.70648764373457 | 2.663359959920247 |
| | 1.7111652142172795 | 1.94877052307131 |
| | -1.66787594106593 | -1.552368799845377 |
| | 2.96875928857484 | 2.761862436930339 |AT4G23150_CRK7
AT2G29490_GSTU1
AT1G30900_VSR6
AT3G57240_BG3
AT1G13470
AT4G30640_RNI
AT1G14880_PCR1
AT4G34380
AT4G18250
AT5G59680
AT2G04515
real-time PCR
RNA-seq data
Log2 fold change
FIGURE S1
