# Supplementary material for: Ectopic expression of Medicago truncatula homeodomain finger protein, MtPHD6, enhances drought tolerance in Arabidopsis
Source: BMC Genomics. 2019 Dec 16;20:982. doi: 10.1186/s12864-019-6350-5 (PMC6916436; doi:10.1186/s12864-019-6350-5)
Supplement: Supplementary file 8 — Additional file 8: Figure S2. GO term enrichment analysis of genes changed by drought or MtPHD6 transgene in Arabidopsis. [file 12864_2019_6350_MOESM8_ESM.pptx]

## Slide 1
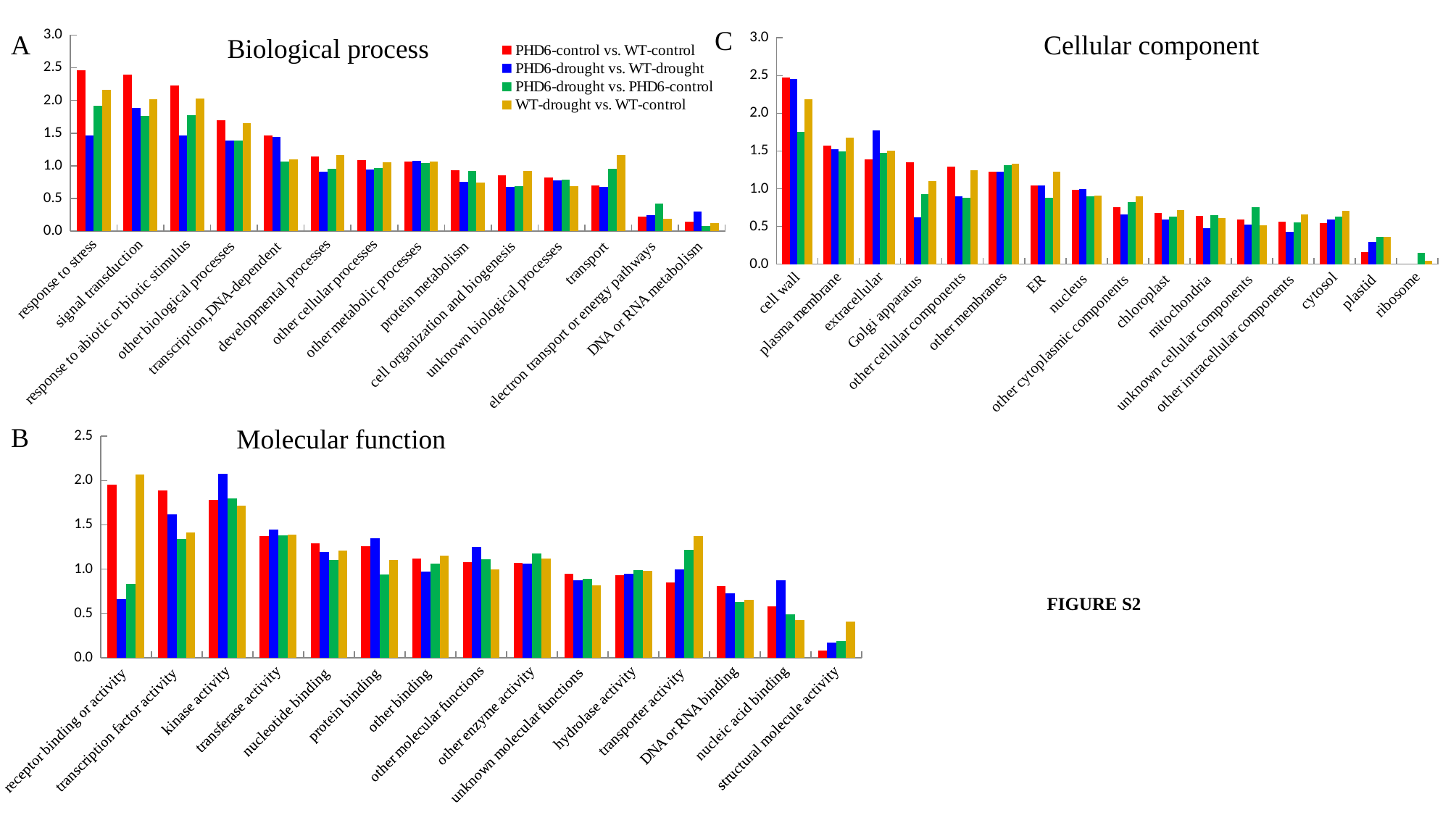

C
A
Cellular component
### Chart
| Category | PHD6-control vs. WT-control | PHD6-drought vs. WT-drought | PHD6-drought vs. PHD6-control | WT-drought vs. WT-control |
|---|---|---|---|---|
| response to stress | 2.46 | 1.46 | 1.9200000000000021 | 2.16 |
| signal transduction | 2.3899999999999997 | 1.8900000000000001 | 1.76 | 2.02 |
| response to abiotic or biotic stimulus | 2.23 | 1.46 | 1.77 | 2.03 |
| other biological processes | 1.7 | 1.3900000000000001 | 1.3900000000000001 | 1.6500000000000001 |
| transcription,DNA-dependent | 1.46 | 1.44 | 1.06 | 1.1 |
| developmental processes | 1.1399999999999941 | 0.91 | 0.9600000000000006 | 1.1599999999999941 |
| other cellular processes | 1.09 | 0.9400000000000006 | 0.9700000000000006 | 1.05 |
| other metabolic processes | 1.07 | 1.08 | 1.04 | 1.07 |
| protein metabolism | 0.93 | 0.760000000000003 | 0.92 | 0.7400000000000027 |
| cell organization and biogenesis | 0.8600000000000007 | 0.68 | 0.6900000000000006 | 0.92 |
| unknown biological processes | 0.8200000000000006 | 0.78 | 0.79 | 0.6900000000000006 |
| transport | 0.7000000000000006 | 0.68 | 0.9600000000000006 | 1.1700000000000021 |
| electron transport or energy pathways | 0.22 | 0.25 | 0.4200000000000003 | 0.19 |
| DNA or RNA metabolism | 0.14 | 0.3000000000000003 | 0.08000000000000004 | 0.12000000000000002 |Biological process
### Chart
| Category | PHD6-control vs. WT-control | PHD6-drought vs. WT-drought | PHD6-drought vs. PHD6-control | WT-drought vs. WT-control |
|---|---|---|---|---|
| cell wall | 2.4699999999999998 | 2.4499999999999997 | 1.75 | 2.19 |
| plasma membrane | 1.57 | 1.52 | 1.49 | 1.6800000000000053 |
| extracellular | 1.3900000000000001 | 1.77 | 1.48 | 1.5 |
| Golgi apparatus | 1.35 | 0.6200000000000027 | 0.93 | 1.1 |
| other cellular components | 1.29 | 0.9 | 0.88 | 1.25 |
| other membranes | 1.23 | 1.23 | 1.31 | 1.33 |
| ER | 1.04 | 1.04 | 0.88 | 1.23 |
| nucleus | 0.99 | 1.0 | 0.9 | 0.91 |
| other cytoplasmic components | 0.760000000000003 | 0.6600000000000034 | 0.8200000000000006 | 0.9 |
| chloroplast | 0.68 | 0.59 | 0.630000000000003 | 0.7200000000000006 |
| mitochondria | 0.640000000000003 | 0.4800000000000003 | 0.6500000000000031 | 0.6100000000000007 |
| unknown cellular components | 0.59 | 0.53 | 0.760000000000003 | 0.52 |
| other intracellular components | 0.56 | 0.4300000000000004 | 0.55 | 0.6600000000000034 |
| cytosol | 0.54 | 0.59 | 0.630000000000003 | 0.7100000000000006 |
| plastid | 0.16 | 0.2900000000000003 | 0.3600000000000003 | 0.3600000000000003 |
| ribosome | None | None | 0.15000000000000024 | 0.04000000000000002 |B
Molecular function
### Chart
| Category | PHD6-control vs. WT-control | PHD6-drought vs. WT-drought | PHD6-drought vs. PHD6-control | WT-drought vs. WT-control |
|---|---|---|---|---|
| receptor binding or activity | 1.9500000000000062 | 0.660000000000004 | 0.8300000000000006 | 2.07 |
| transcription factor activity | 1.8900000000000001 | 1.62 | 1.34 | 1.41 |
| kinase activity | 1.78 | 2.08 | 1.8 | 1.72 |
| transferase activity | 1.37 | 1.45 | 1.3800000000000001 | 1.3900000000000001 |
| nucleotide binding | 1.29 | 1.1900000000000062 | 1.1 | 1.21 |
| protein binding | 1.26 | 1.35 | 0.9400000000000006 | 1.1 |
| other binding | 1.12 | 0.9700000000000006 | 1.06 | 1.149999999999993 |
| other molecular functions | 1.08 | 1.25 | 1.11 | 1.0 |
| other enzyme activity | 1.07 | 1.06 | 1.1800000000000062 | 1.12 |
| unknown molecular functions | 0.9500000000000006 | 0.8700000000000031 | 0.89 | 0.8200000000000006 |
| hydrolase activity | 0.93 | 0.9500000000000006 | 0.99 | 0.98 |
| transporter activity | 0.8500000000000006 | 1.0 | 1.22 | 1.37 |
| DNA or RNA binding | 0.81 | 0.7300000000000006 | 0.6300000000000034 | 0.6500000000000037 |
| nucleic acid binding | 0.5800000000000001 | 0.8700000000000031 | 0.4900000000000003 | 0.4200000000000003 |
| structural molecule activity | 0.08000000000000004 | 0.17 | 0.19 | 0.4100000000000003 |FIGURE S2
