# Supplementary material for: Ectopic expression of Medicago truncatula homeodomain finger protein, MtPHD6, enhances drought tolerance in Arabidopsis
Source: BMC Genomics. 2019 Dec 16;20:982. doi: 10.1186/s12864-019-6350-5 (PMC6916436; doi:10.1186/s12864-019-6350-5)
Supplement: Supplementary file 9 — Additional file 9: Figure S3. Cluster and network analyses of AP2/EREBP and WRKY transcription factors. For cluster analysis, the list of genes were analyzed using Cluster 3.0 and the resulting tree figure was shown by Java Treeview. Network analysis was performed using STRING (https://string-db.org) and Cytoscape 3.7.1. a: Cluster analysis of AP2/EREBP transcription factors affected by MtPHD6 transgene, drought and ABA. b and c: Networks of AP2/EREBP transcription factors modulated by drought stress (green node and red edge) and MtPHD6 transgene (red node and green edge), respectively. d: Cluster analysis of WRKY transcription factors affected by MtPHD6 transgene, drought and ABA. e and f: Networks of WRKY transcription factors modulated by drought stress (green node and red edge) and MtPHD6 transgene (red node and green edge), respectively. The original data were presented in Additional file 6: Table S6. ABA treatment expression data were downloaded from BAR Expression Browser (http://bar.utoronto.ca/affydb/cgi-bin/affy_db_exprss_browser_in.cgi). The grey color in cluster analysis indicates the genes are missing in BAR database. [file 12864_2019_6350_MOESM9_ESM.pptx]

## Slide 1
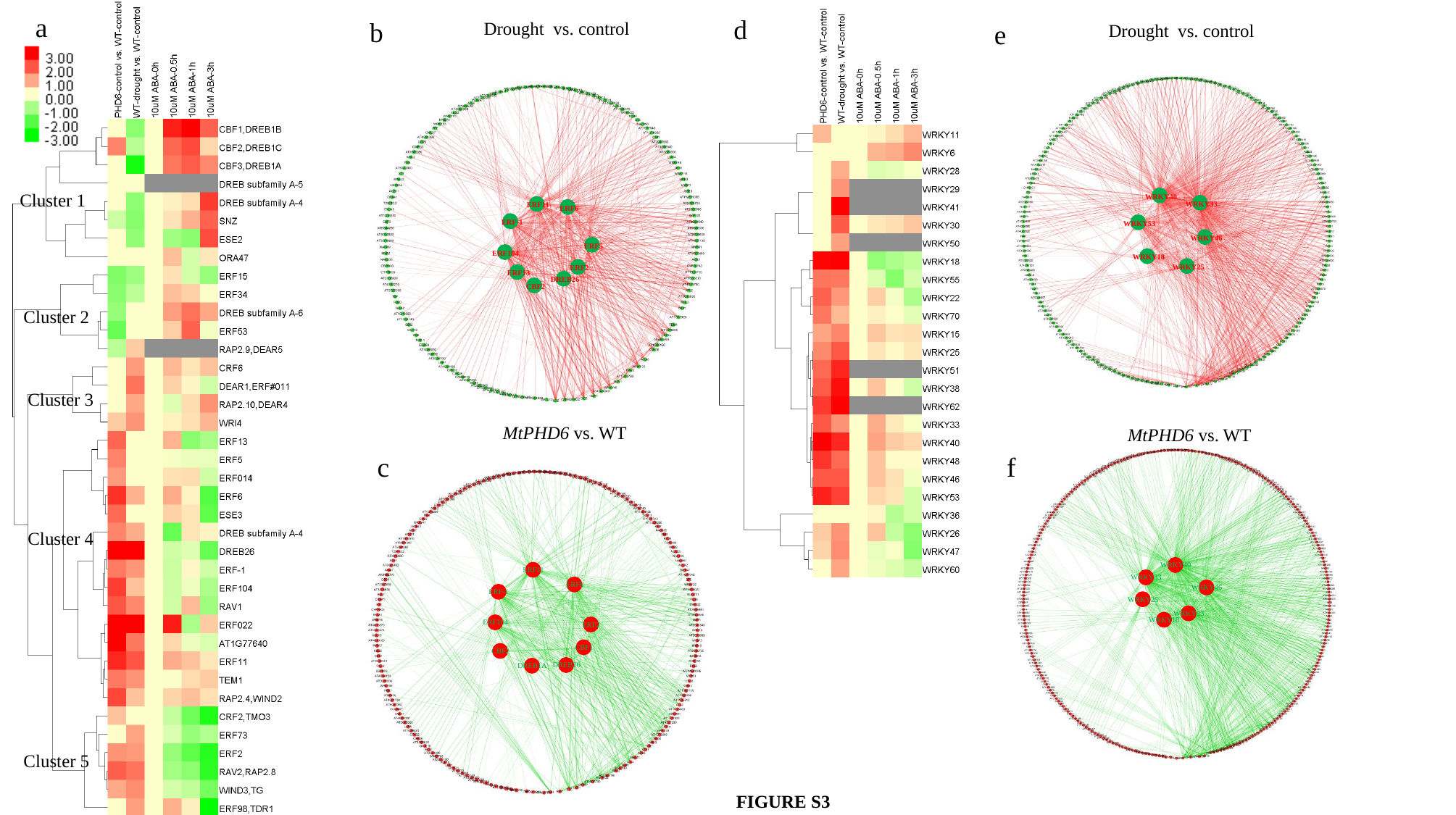

a
d
b
Drought vs. control
e
Drought vs. control
Cluster 1
WRKY40
WRKY33
ERF11
ERF6
ERF-1
WRKY53
WRKY46
ERF5
ERF104
WRKY18
WRKY25
ERF2
ERF13
DREB26
CBF2
Cluster 2
Cluster 3
MtPHD6 vs. WT
MtPHD6 vs. WT
c
f
Cluster 4
WRKY40
ERF11
WRKY33
ERF6
WRKY46
ERF-1
WRKY25
WRKY53
WRKY18
ERF104
ERF2
CBF1
CBF2
DREB26
DREB1A
Cluster 5
FIGURE S3
